# Trained immunity suppression determines kidney allograft survival

**DOI:** 10.1016/j.ajt.2024.08.006

**Published:** 2024-08-13

**Authors:** Inge Jonkman, Maaike M.E. Jacobs, Yutaka Negishi, Cansu Yanginlar, Joost H.A. Martens, Marijke Baltissen, Michiel Vermeulen, Martijn W.F. van den Hoogen, Marije Baas, Johan van der Vlag, Zahi A. Fayad, Abraham J.P. Teunissen, Joren C. Madsen, Jordi Ochando, Leo A.B. Joosten, Mihai G. Netea, Willem J.M. Mulder, Musa M. Mhlanga, Luuk B. Hilbrands, Nils Rother, Raphaël Duivenvoorden

**Affiliations:** 1 Department of Nephrology, Radboud University Medical Center, Nijmegen, The Netherlands; 2 Department of Molecular Biology, Faculty of Science, Oncode Institute, Radboud University Nijmegen, Nijmegen, The Netherlands; 3 Department of Cell Biology, Faculty of Science, Radboud University Nijmegen, Nijmegen, The Netherlands; 4 Department of Internal Medicine, Erasmus Medical Center Transplant Institute, University Medical Center Rotterdam, Rotterdam, The Netherlands; 5 Department of Radiology, Biomolecular Engineering and Imaging Institute, Icahn School of Medicine at Mount Sinai, New York, New York, USA; 6 Department of Surgery, Center for Transplantation Sciences, Massachusetts General Hospital, Boston, Massachusetts, USA; 7 Division of Cardiac Surgery, Department of Surgery, Massachusetts General Hospital, Boston, Massachusetts, USA; 8 Department of Oncological Sciences, Icahn School of Medicine at Mount Sinai, New York, New York, USA; 9 Transplant Immunology Unit, National Center of Microbiology, Instituto de Salud Carlos III, Madrid, Spain; 10 Department of Internal Medicine and Radboud Center for Infectious Diseases, Radboud University Medical Center, Nijmegen, The Netherlands; 11 Department of Medical Genetics, University of Medicine and Pharmacy, Iuliu Haţieganu, Cluj-Napoca, Romania; 12 Department of Immunology and Metabolism, Life and Medical Sciences Institute, University of Bonn, Bonn, Germany; 13 Department of Biomedical Engineering and Institute for Complex Molecular Systems, Laboratory of Chemical Biology, Eindhoven University of Technology, Eindhoven, The Netherlands

**Keywords:** trained immunity, innate immunity, kidney transplantation, graft survival

## Abstract

The innate immune system plays an essential role in regulating the immune responses to kidney transplantation, but the mechanisms through which innate immune cells influence long-term graft survival are unclear. The current study highlights the vital role of trained immunity in kidney allograft survival. Trained immunity describes the epigenetic and metabolic changes that innate immune cells undergo following an initial stimulus, allowing them have a stronger inflammatory response to subsequent stimuli. We stimulated healthy peripheral blood mononuclear cells with pretransplant and posttransplant serum of kidney transplant patients and immunosuppressive drugs in an in vitro trained immunity assay and measured tumor necrosis factor and interleukin 6 cytokine levels in the supernatant as a readout for trained immunity. We show that the serum of kidney transplant recipients collected 1 week after transplantation can suppress trained immunity. Importantly, we found that kidney transplant recipients whose serum most strongly suppressed trained immunity rarely experienced graft loss. This suppressive effect of posttransplant serum is likely mediated by previously unreported effects of immunosuppressive drugs. Our findings provide mechanistic insights into the role of innate immunity in kidney allograft survival, uncovering trained immunity as a potential therapeutic target for improving graft survival.

## 1. Introduction

Kidney transplantation is a life-saving procedure for patients with kidney failure. To prevent graft rejection, patients need to use potent immunosuppressive drugs.^1^ Although therapeutic developments over the past decades have reduced the risk of acute rejection, little improvement in long-term graft survival has been realized.^2^ A clear medical need exists for new therapeutic strategies to improve long-term outcomes, but this requires insight into how the immune system can be effectively targeted.

Currently used immunosuppressive drugs in kidney transplantation are primarily applied to suppress the adaptive immune system. T and B cells are crucial in orchestrating the immune response against foreign antigens from the transplanted graft.^3^ Nevertheless, innate immune cells also play an important role in mediating graft rejection, as demonstrated by preclinical and clinical studies.^4–12^ Moreover, innate immune cells are involved in nonallogenic mechanisms of tissue damage that determine long-term graft survival. Ischemia-reperfusion injury (IRI) induces the activation of a damaging innate immune response.^13–15^ Innate immune cells also promote interstitial fibrosis and vasculopathy, leading to chronic kidney allograft disease.^16,17^ This makes the innate immune system a promising target for promoting graft survival.

Trained immunity is of particular interest in the context of organ transplantation.^18^ Trained immunity refers to a phenomenon in which innate immune cells can acquire long-term immunologic memory after exposure to microorganisms or endogenous inflammatory signals. This memory is maintained by metabolic and epigenetic changes that result in enhanced inflammatory responses to restimulation.^19,20^ Trained immunity can be induced peripherally in blood or tissue and harbors within the hematopoietic stem cell niche of the bone marrow. The latter enables durable production of myeloid cells with a trained (ie, hyperresponsive) phenotype.^21^ Although often beneficial in infections, inappropriate induction of trained immunity can also promote damaging inflammation, including in atherosclerosis, auto-inflammatory diseases, and organ transplantation.^18^

Here, we investigated trained immunity induction in human kidney transplant recipients and evaluated its impact on graft survival. We found that serum obtained from kidney transplant recipients 1 week after transplantation effectively suppresses trained immunity in cellular assays. Importantly, the degree of trained immunity suppression was independently associated with improved graft survival. Using comprehensive in vitro and in vivo analyses, we examined if sterile inflammation induced by IRI or the use of immunosuppressive drugs could explain the suppressive effect of posttransplant serum on trained immunity. We additionally investigated how trained immunity-regulating properties correlate with epigenetic and transcriptomic changes in these patient’s circulating leukocytes. Our results highlight the importance of trained immunity induction in regulating graft survival and its potential as a therapeutic target for improving outcomes.

## 2. Methods

### 2.1. Mouse studies

We used 8- to 12-week-old C57BL/6J mice in which we induced unilateral renal ischemia for 30, 35, and 40 minutes or performed a sham procedure (n = 6 per group). We sacrificed 50% of the mice (n = 3) after 3 days and the remaining mice (n = 3) after 7 days and collected their spleen, bone marrow, and kidneys. We performed flow cytometry on kidney immune cells (Animal Ethical Committee of the Radboud University Nijmegen, approval number AVD1030020198545).

### 2.2. Human studies

For in vitro studies on human peripheral blood mononuclear cells (PBMCs), buffy coats from healthy donors were obtained from Sanquin blood bank, Nijmegen after obtaining written consent. Serum and PBMCs of transplant patients collected before and 1 week after kidney transplantation were obtained from patients included in a randomized controlled trial conducted at Radboud University Medical Center.^22,23^ All patients provided written informed consent before study entry. The study was approved by the Committee on Human-Related Research Arnhem–Nijmegen and conducted according to the Declaration of Helsinki and good clinical practice guidelines (NCT00565331, EudraCT number: 2007-001604-20).

Trained immunity assays were performed by incubating healthy donor PBMCs with kidney transplant recipient’s serum, danger-associated molecular patterns (DAMPs), or immunosuppressive drugs for 24 hours followed by a 5-day rest period and 24-hour restimulation with lipopolysaccharide (LPS). Induction of trained immunity was assessed by measuring interleukin (IL)-6 and tumor necrosis factor (TNF) cytokine production in the supernatant with an enzyme-linked immunosorbent assay. IL-6 levels in human serum were measured with the Ella Automated Immunoassay System (Bio-techne). Kidney transplant recipient’s serum markers of inflammation and tissue damage were measured with the Olink Proximity Extension Assay technology (Olink Target 96 Inflammation panel, Olink Bioscience). We performed whole-genome ribonucleic acid sequencing (RNA-seq) and whole-genome assessment of histone 3 lysine 4 trimethylation (H3K4me3) and histone 3 lysine 27 acetylation (H3K27ac) by chromatin immunoprecipitation sequencing (ChIP-seq) on PBMCs obtained 1 week after transplantation from 12 kidney transplant recipients.

### 2.3. Statistics

Continuous data are presented as mean with standard deviation (SD) or standard error of the mean. In case of nonnormal distribution of the data, medians and interquartile ranges are reported. Categorical data are presented as percentages. Unpaired *t* test, paired *t* test, 1-way analysis of variance, and Mann-Whitney *U* tests were used to assess between group differences. Death-censored graft survival was assessed using the Kaplan-Meier method, and between group differences were determined using a log-rank test. Cox proportional hazard regression analyses were performed to identify independent risk factors for death-censored graft survival. Multivariable regression models were obtained using a backward elimination procedure. Variables were retained if *P* <.05. Analyses were performed with the statistical software IBM SPSS Statistics for Windows (version 27.0; IBM Corp). A *P* value < .05 indicated statistical significance. All other information is detailed in Supplementary Methods.

## 3. Results

### 3.1. The effect of human kidney transplant recipient’s serum on trained immunity

Serum was obtained before and 1 week after kidney transplantation from 96 patients included in a randomized controlled trial in which we studied the efficacy and safety of rituximab as induction therapy after kidney transplantation.^22,23^ Baseline characteristics are shown in Supplementary Table 1. The mean age of the kidney transplant recipients was 50.4 (SD, 14.0) years, 30% were female, 23% of patients underwent pre-emptive kidney transplantation, and 54% of transplanted kidneys were derived from living donors. The mean follow-up during the study was 6.8 (SD, 2.4) years.

**Figure 1.**
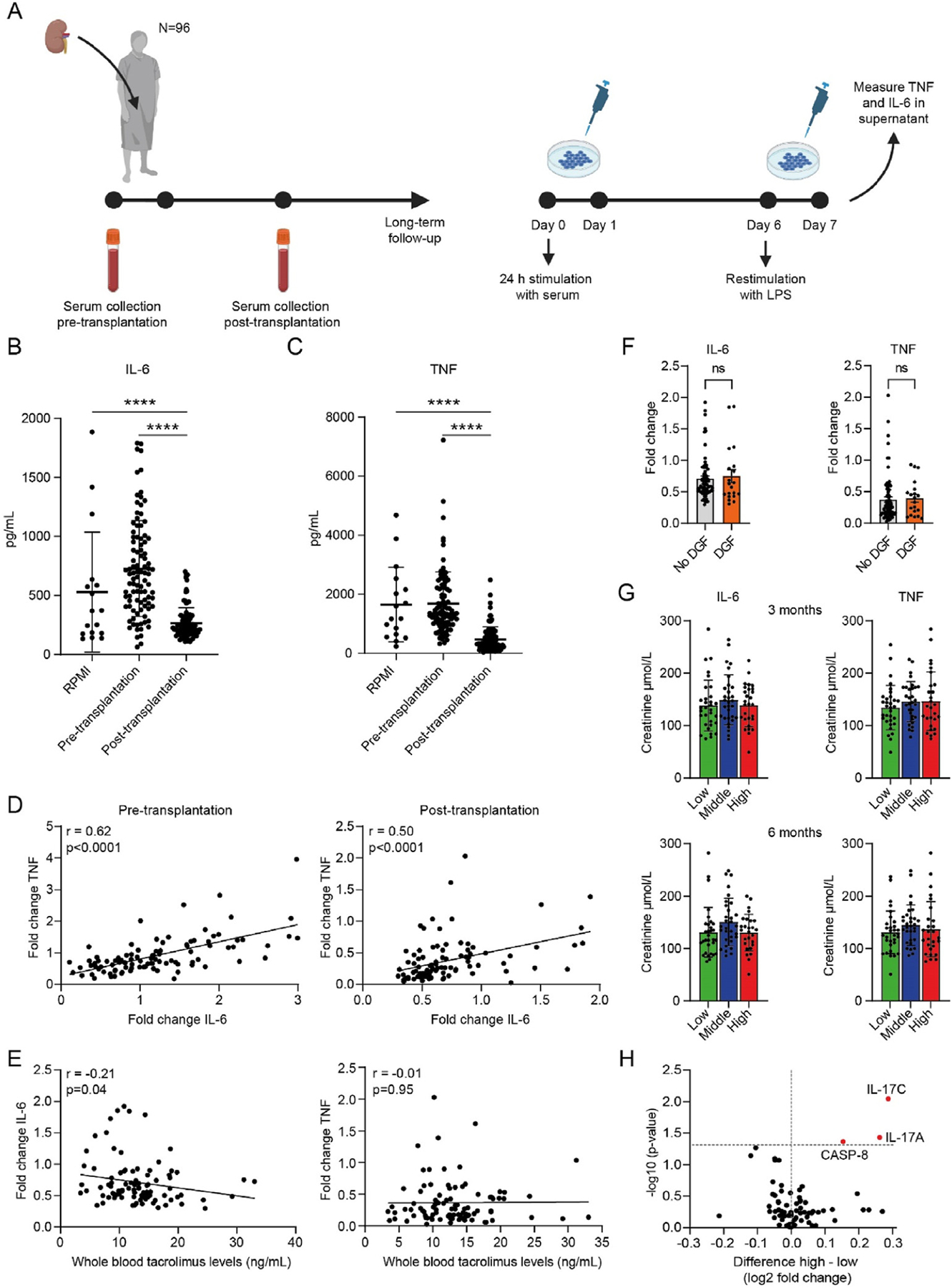
The effect of human kidney transplant recipient’s serum on trained immunity. (A) Schematic representation of serum collection and the subsequent serum-trained immunity assay. (B, C) PBMCs were incubated for 24 hours with pre- and posttransplant serum of 96 kidney transplant recipients. After a 5-day resting period, cells were restimulated with LPS for 24 hours, and IL-6 and TNF cytokine production was measured in the supernatant by ELISA (n = 3). RPMI culture medium without patient serum was used as a negative control. (D) Correlation between IL-6 and TNF levels measured in the supernatant after serum-trained immunity assay with pre- and posttransplant serum of 96 kidney transplant recipients. (E) Correlation between IL-6 and TNF levels measured in the supernatant after posttransplant serum-trained immunity assay of 96 kidney transplant recipients and their whole blood tacrolimus levels posttransplant. (F) Comparison between IL-6 and TNF response in cells trained with posttransplant serum from patients with DGF compared to patients with no DGF. (G) Comparison of tertiles of IL-6 and TNF responses from cells trained with posttransplant serum and creatinine levels at 3 and 6 months after transplantation. (H) Volcano plot of 76 inflammation-related proteins (Olink inflammation panel) measured in posttransplant serum of the highest tertile compared to the lowest tertile of the IL-6 and TNF values in the trained immunity assay. Data are expressed as mean ± SD (B, C, G) or as mean ± SEM (F). Fold changes were calculated as value/mean RPMI (D, E). *P* values were calculated using unpaired *t* test (B, C, F), 1-way analysis of variance (G), or Mann-Whitney *U* test (H). ****P < .0001. DGF, delayed graft function; ELISA, enzyme-linked immunosorbent assay; IL-6; inter-leukin 6; LPS, lipopolysaccharide; PBMC, peripheral blood mononuclear cell; SD, standard deviation; SEM, standard error of the mean; TNF, tumor necrosis factor; CASP-8, caspase-8.

PBMCs of healthy subjects were incubated with patient serum for 24 hours, followed by a 5-day rest period and 24-hour restimulation with LPS or RPMI medium as a negative control (Fig. 1A). Trained immunity was assessed by measuring IL-6 and TNF cytokine production in the supernatant with enzyme-linked immunosorbent assay. The IL-6 and TNF responses of cells trained with pretransplant serum showed considerable heterogeneity (Fig. 1B, C). Using a multivariate linear regression analysis with backward elimination, we found that the kidney transplant recipient’s age, body mass index, and pretransplant IL-6 serum level were associated with the IL-6 response, whereas the etiology of kidney failure correlated with the TNF response to LPS restimulation (Supplementary Table 2).

Compared to cells trained with pretransplant serum, the IL-6 and TNF responses to LPS restimulation of cells trained with posttransplant serum were substantially lower, with a mean difference of 463 pg/mL (SD, 428 pg/mL; *P* < .001) for the IL-6 response and 1215 pg/mL (SD, 1078 pg/mL; *P* < .001) for the TNF response to LPS restimulation (Fig. 1B, C, Supplementary Fig. S1). IL-6 and TNF responses correlated strongly in the cells trained with pretransplant serum and those trained with posttransplant serum (Fig. 1D).

In a multivariate linear regression analysis with backward elimination, we found no association of the posttransplant trained immunity response with clinical parameters. The IL-6 response between cells trained with posttransplant serum negatively correlated with tacrolimus whole blood concentrations in the first week after transplantation (Spearman’s ρ −0.21, *P* = .04) (Fig. 1E). This was not the case for posttransplant serum tacrolimus levels (Supplementary Fig. S2). Posttransplant serum IL-6 levels did not correlate with the trained immunity response (Supplementary Fig. S3).

We found no difference in the IL-6 or TNF response of cells trained with posttransplant serum obtained from patients with vs patients without delayed graft function (Fig. 1F; Supplementary Fig. S4). We then divided the 96 patients in tertiles based on the posttransplant serum-induced trained immunity IL-6 and TNF responses. We found no differences in creatinine levels at 3 and 6 months after transplantation (Fig. 1G).

We explored if the posttransplant serum-trained immunity response was associated with markers of inflammation and tissue damage in posttransplant serum using the Olink Proximity Extension Assay technology (Olink Bioscience). We compared serum protein levels between the lowest and highest tertiles of the IL-6 and TNF trained immunity responses. Overall, we found little differences in serum markers, except for IL-17C, IL-17A, and CASP8, which were increased in the highest tertile (Fig. 1H).

### 3.2. Trained immunity determines kidney allograft survival

To investigate the association of serum-induced trained immunity with clinical outcomes, we divided the 96 patients into tertiles according to the IL-6 and TNF responses to LPS restimulation of cells trained with pretransplant and posttransplant serum. Baseline characteristics according to tertiles of pretransplant and posttransplant IL-6 and TNF responses are shown in Supplementary Tables 3–6. Baseline characteristics were largely comparable between tertiles.

We performed Kaplan-Meier survival analyses using the log-rank test to assess the association between the IL-6 and TNF tertiles and the occurrence of biopsy-proven acute rejection (BPAR), which was documented in the first 2 years after transplantation. We found no differences in the occurrence of BPAR among the tertiles (Supplementary Figs. S5 and S6).

**Figure 2.**
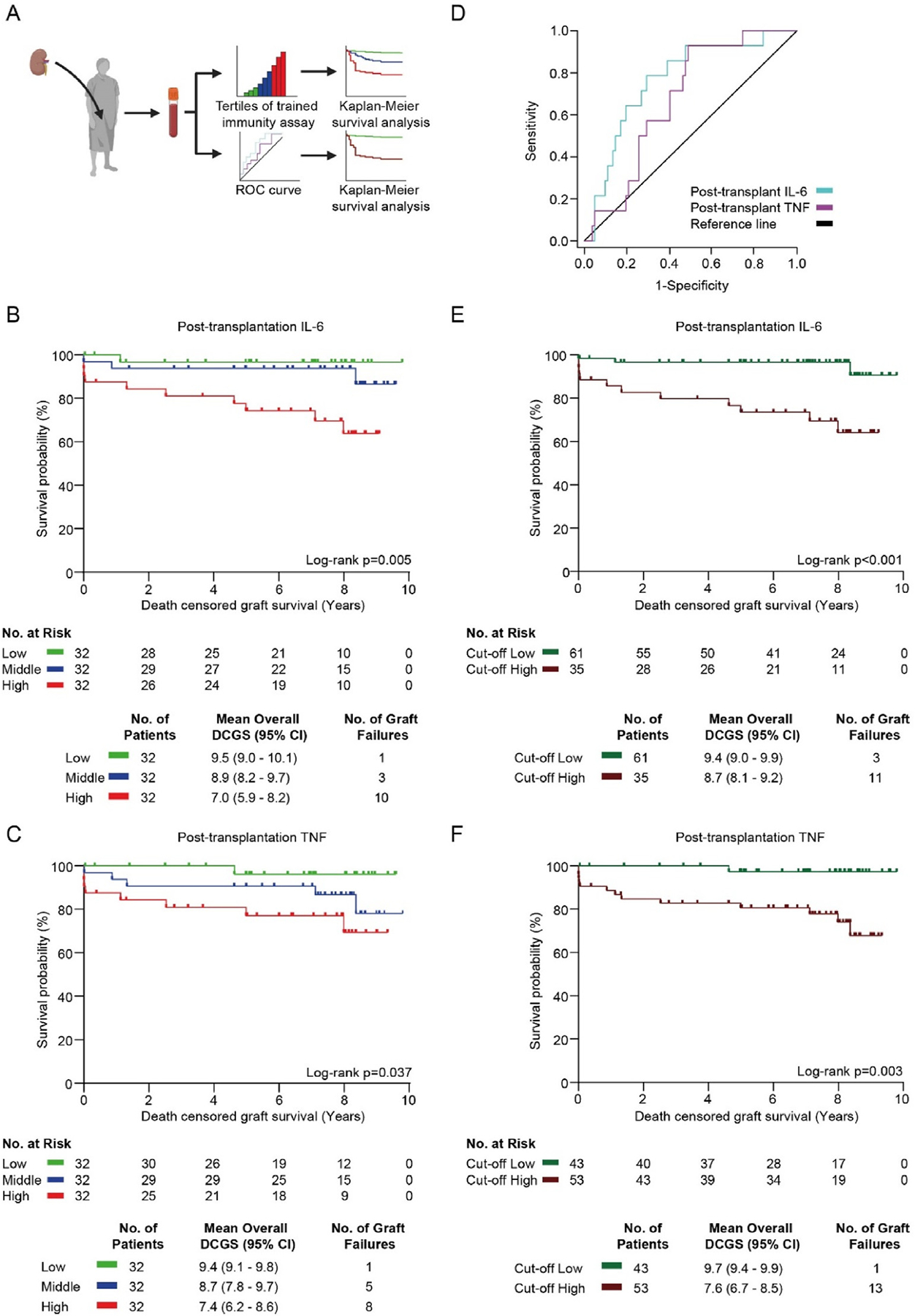
Trained immunity is associated with long-term graft survival. (A) Schematic representation of the method of collecting the data for Kaplan-Meier survival analyses. Results of serum-induced trained immunity were divided into tertiles (low = green, middle = blue, and high = red) or by cutoffs determined based on the ROC curves. (B, C) Kaplan-Meier survival analyses of death-censored graft survival of the tertiles of serum-induced trained immunity for the IL-6 and TNF responses to LPS restimulation. Data are expressed as survival probability (%) and death-censored graft survival (y). *P* values were calculated with a log-rank test. (D) ROC curves for the IL-6 and TNF responses to LPS restimulation. (E, F) Kaplan-Meier survival analyses of death-censored graft survival of the low vs the high posttransplant serum-induced trained immunity for the IL-6 and TNF responses to LPS restimulation, based on the cutoffs determined by the ROC curves. Data are expressed as survival probability (%) and death-censored graft survival (years). *P* values were calculated with a log-rank test. CI, confidence interval; DCGS, death-censored graft survival; IL-6; interleukin 6; LPS, lipopolysaccharide; ROC, receiver operating characteristic; TNF, tumor necrosis factor.

Next, we investigated death-censored graft survival in the tertiles of the IL-6 and TNF responses to LPS restimulation (Fig. 2A). For the posttransplant IL-6 response, the mean death-censored graft survival was 9.5 years (95% confidence interval [CI], 9.0–10.1), 8.9 years (95% CI, 8.2–9.7), and 7.0 years (95% CI, 5.9–8.2) in the lowest, middle and highest tertiles, respectively. Death-censored graft survival was 96.9%, 90.6%, and 68.8% for the lowest, middle, and highest tertiles, respectively (Fig. 2B). For the posttransplant TNF response, the mean death-censored graft survival was 9.4 years (95% CI, 9.1–9.8), 8.7 years (95% CI, 7.8–9.7), and 7.4 years (95% CI, 6.2–8.6) in the lowest, middle and highest tertiles, respectively. Death-censored graft survival was 96.9%, 84.4%, and 75.0% for the lowest, middle, and highest tertiles, respectively (Fig. 2C). Using Cox proportional-hazards models with a backward elimination procedure, we identified predictors of death-censored graft survival in which variables with *P* < .1 were selected as predictors. The tertiles of the IL-6 and TNF responses were independent predictors of death-censored graft survival (Supplementary Tables 7 and 8). We did not observe any differences in death-censored graft survival in the pretransplant IL-6 and TNF responses among the tertiles (Supplementary Fig. S7).

To investigate how the posttransplant IL-6 and TNF responses discriminate kidney transplant recipients that experience graft loss from those with long-term graft survival, we performed receiver operating characteristic (ROC) curve analyses. The ROC curves showed a better performance of the IL-6 response than the TNF response (area under the curve 0.77 and 0.68, respectively; Fig. 2D). The ROC curves indicated that a posttransplant IL-6 response of 252 pg/mL is the best cutoff value to discriminate between patients with a low vs high death-censored graft survival rate, yielding a sensitivity of 78.6% and a specificity of 70.7% (Fig. 2D). For the TNF response, a cutoff of 293 pg/mL yields a sensitivity of 92.9% and a specificity of 51.2% (Fig. 2D). Using these cutoffs, we made Kaplan-Meier survival plots showing the cumulative death-censored graft survival probability (Fig. 2E, F). For the IL-6 response, death-censored graft survival was 95.1% vs 68.6% in the groups below and above the cutoff value, respectively (Fig. 2E). For the TNF response, death-censored graft survival was 97.7% vs 75.5% in the groups below and above the cutoff value, respectively (Fig. 2F). Trained immunity’s associations with graft survival were similar in patients who received a kidney from a living vs deceased donor (Supplementary Figs. S8–S10).

### 3.3. The effect of sterile inflammation on trained immunity

**Figure 3.**
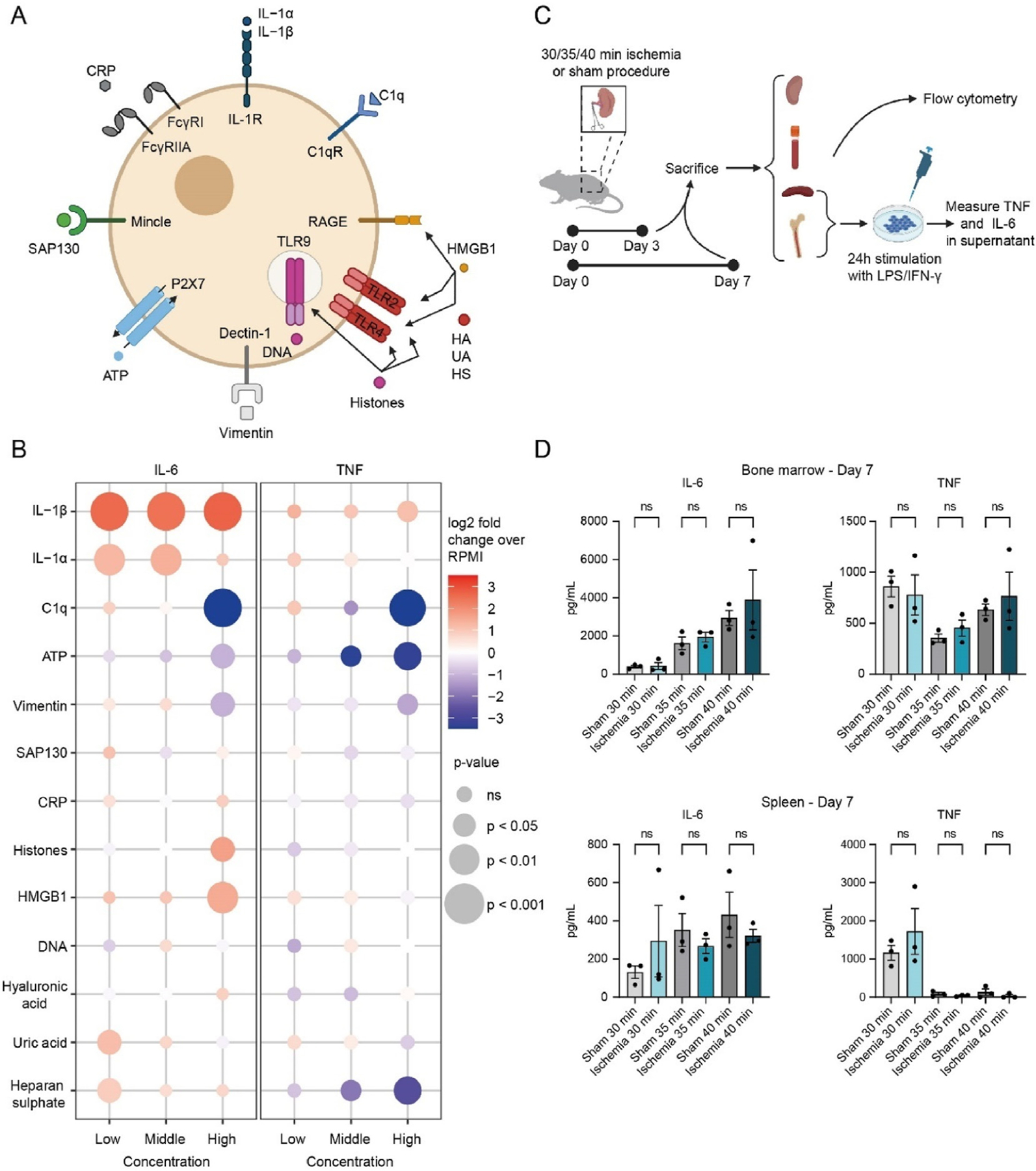
The in vitro and ex vivo effects of sterile inflammation on trained immunity. (A) Schematic representation indicating trained immunity-related DAMPs and their respective receptors. (B) PBMCs were stimulated for 24 hours with DAMPs in 3 concentrations. After a 5-day resting period, cells were restimulated with LPS for 24 hours, and IL-6 and TNF were quantified in the supernatant by ELISA (n = 6). (C) Schematic representation of the mouse ischemia model, subsequent flow cytometry, and ex vivo stimulation assay. (D) Unilateral renal ischemia was induced in 8- to 12-week-old C57BL/6J mice for 30, 35, or 40 minutes. Sham mice underwent the same procedure without clamping the renal artery. Mice were sacrificed on day 3, and BMDMs and splenocytes were collected and stimulated for 24 hours with LPS or culture medium (DMEM:HAMF12) as a control before collecting the supernatant. IL-6 and TNF were quantified by ELISA (n = 3). Data are expressed as log2 fold change compared to untrained (RPMI) PBMCs (A) or as mean ± SEM (B, D). *P* values were calculated using an unpaired *t* test (A) or a paired *t* test (B, D). BMDM, bone marrow-derived macrophage; DAMP, danger-associated molecular pattern; ELISA, enzyme-linked immunosorbent assay; HMGB1, high mobility group box 1 protein; IFN, interferon; IL; interleukin; LPS, lipopolysaccharide; PBMC, peripheral blood mononuclear cell; SEM, standard error of the mean; TNF, tumor necrosis factor; CRP, C-reactive protein; SAP130, Sin3A associated protein; ATP, adenosine triphosphate; RAGE, Receptor for Advanced Glycation Endproducts; TLR, Toll-like receptor; HA, hyaluronic acid; UA, uric acid; HS, heparan sulphate; ns, not significant.

Because we found that serum from kidney transplant recipients after transplantation has a suppressive effect on the trained immune response, we investigated if this could be caused by DAMPs and inflammatory cytokines released during transplantation. To test this, we studied a panel of 13 DAMPs selected based on the pattern recognizing receptors with which they interact (Fig. 3A).^24,25^ All molecules were tested at nontoxic concentrations (Supplementary Fig. S11). We adapted a previously described in vitro trained immunity protocol in which human PBMCs are stimulated for 24 hours followed by a 5-day resting period and a subsequent 24-hour incubation with LPS, after which we measured IL-6 and TNF production in the supernatant (Supplementary Fig. S12).^26^ We used heat-killed *Candida albicans* (HKCA) as a positive control for trained immunity induction and/or RPMI medium as a negative control.

We observed that stimulation of the IL-1 receptor by IL-1α or IL-1β induces trained immunity and results in a particularly strong IL-6 response upon LPS restimulation. Histones and high mobility group box 1 protein also exacerbated the IL-6 response. However, DNA did not result in elevated TNF or IL-6 responses. Uric acid crystals induced trained immunity for the IL-6 response at its lowest concentration (Fig. 3B, Supplementary Fig. S13). Complement 1q, adenosine triphosphate (ATP), and vimentin showed a suppressive effect on trained immunity (Fig. 3B, Supplementary Fig. S13). Heparan sulfate suppressed the TNF response at high concentrations, whereas at low concentrations, it increased the IL-6 response. C-reactive protein, and Sin3A Associated Protein 130 did not induce trained immunity (Fig. 3B, Supplementary Fig. S13). Serum IL-1β did not correlate with the posttransplant serum-trained immunity response (Supplementary Fig. S14).

Next, we determined the net effect of IRI on trained immunity in vivo. We used 8- to 12-week-old C57BL/6J mice in which we induced unilateral renal ischemia with durations of 30, 35, and 40 minutes or performed the same procedure without clamping the renal artery (sham, n = 6 per renal ischemia duration). We sacrificed 50% of the mice (n = 3) after 3 days and the remaining mice (n = 3) 7 days after IRI induction and collected their spleen, bone marrow, and kidneys. We performed flow cytometry on kidney cells to study the infiltration of immune cells in response to IRI (Supplementary Fig. S15). To investigate the effect of IRI on trained immunity, we stimulated splenocytes or bone marrow cells for 24 hours with LPS, interferon (IFN)-γ, or DMEM:HAMF12 culture medium as negative control. By comparing IL-6 and TNF production by cells obtained from sham mice and mice undergoing kidney IRI, we assessed trained immunity (Fig. 3C). Although we observed neutrophil infiltration in the kidneys after IRI (Supplementary Fig. S15), we saw no evidence of trained immunity in splenocytes and bone marrow-derived cells (Fig. 3D, Supplementary Figs. S16 and S17).

### 3.4. In vitro effect of immunosuppressive drugs on trained immunity

**Figure 4.**
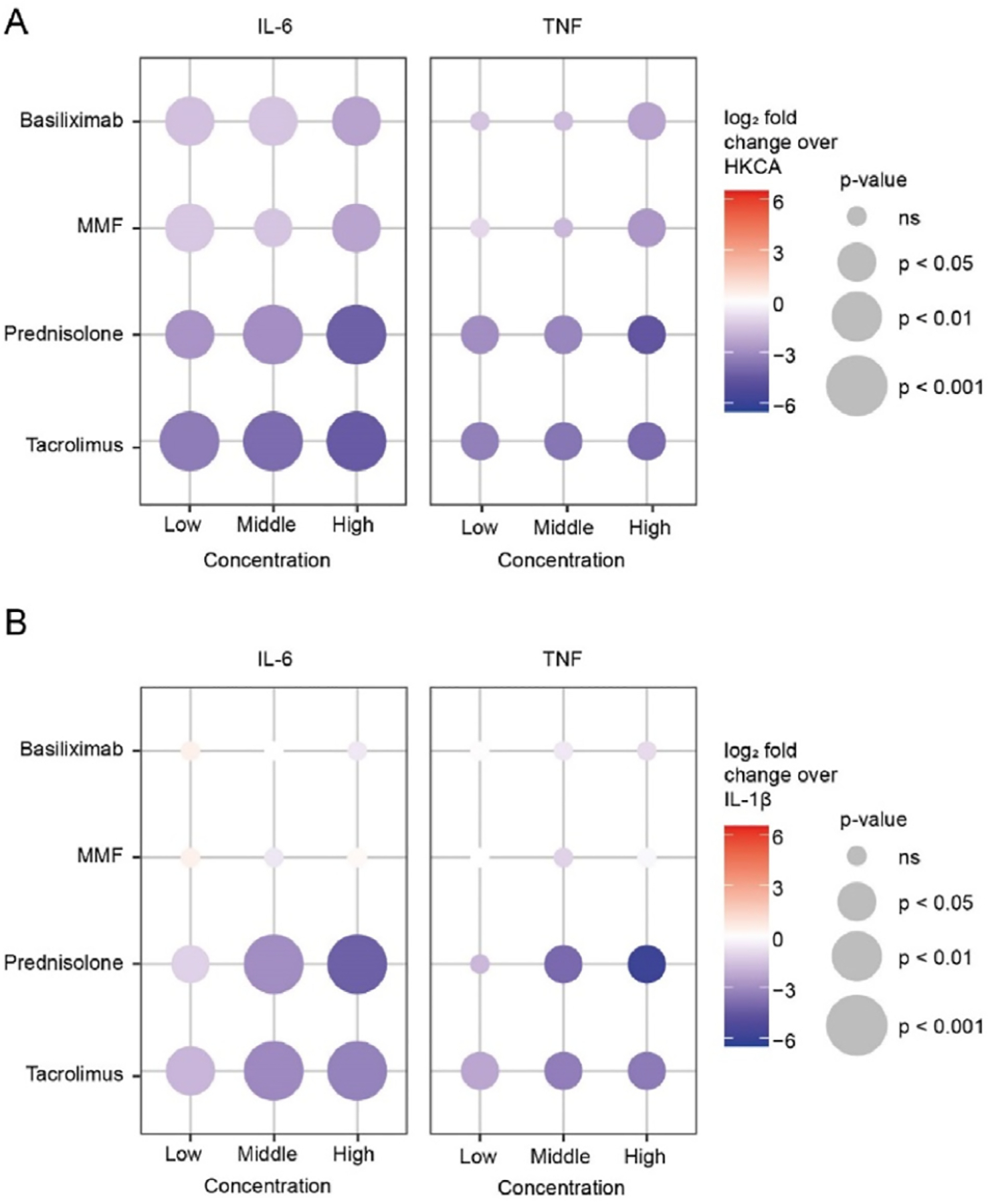
The in vitro effect of immunosuppressive drugs on the induction of trained immunity. (A, B) PBMCs were stimulated for 24 hours with HKCA (A) or IL-1β (B) alone or together with immunosuppressive drugs in 3 different concentrations for 24 hours. After 24 hours, both the stimulus and the immunosuppressive drugs were washed away. After a 5-day resting period, cells were restimulated with LPS for 24 hours, and IL-6 and TNF cytokine production was measured in the supernatant by ELISA (n = 6). Data are expressed as log2 fold change compared to PBMCs trained with either HKCA or IL-1β. *P* values were calculated using an unpaired *t* test. ELISA, enzyme-linked immunosorbent assay; HKCA, heat-killed *Candida albicans*; IL; interleukin; LPS, lipopolysaccharide; PBMC, peripheral blood mononuclear cell; TNF, tumor necrosis factor; ns, not significant.

In organ transplantation, patients receive immunosuppressive drugs to prevent acute rejection. We studied the effects of basiliximab, mycophenolate mofetil, prednisolone, and tacrolimus. All drugs were tested at nontoxic concentrations and in a dose range that resembles plasma concentrations in human kidney transplant recipients (Supplementary Fig. S18). In the assay, we washed away the training stimulus (HKCA or IL-1β) and the drugs after 24 hours. After a 5-day rest period, cells were restimulated with LPS for 24 hours. Subsequently, we quantified IL-6 and TNF in the supernatant. None of the immunosuppressive drugs individually suppressed TNF or IL-6 production after LPS restimulation, whereas combinations of drugs did (Supplementary Fig. S19). Next, we tested whether these immunosuppressive drugs could inhibit the induction of trained immunity by HKCA or IL-1β. Tacrolimus and prednisolone consistently suppressed HKCA- and IL-1β-induced trained immunity (Fig. 4A, B, Supplementary Figs. S20 and S21). Basiliximab and mycophenolate mofetil also suppressed the trained immune response to some extent in HKCA-trained cells but not IL-1β-trained cells. Mostly, tacrolimus and prednisolone inhibited the induction of trained immunity by pretransplant serum (Supplementary Fig. S22).

### 3.5. Transcriptional and epigenetic profiles of the circulating leukocytes of kidney transplant recipients

**Figure 5.**
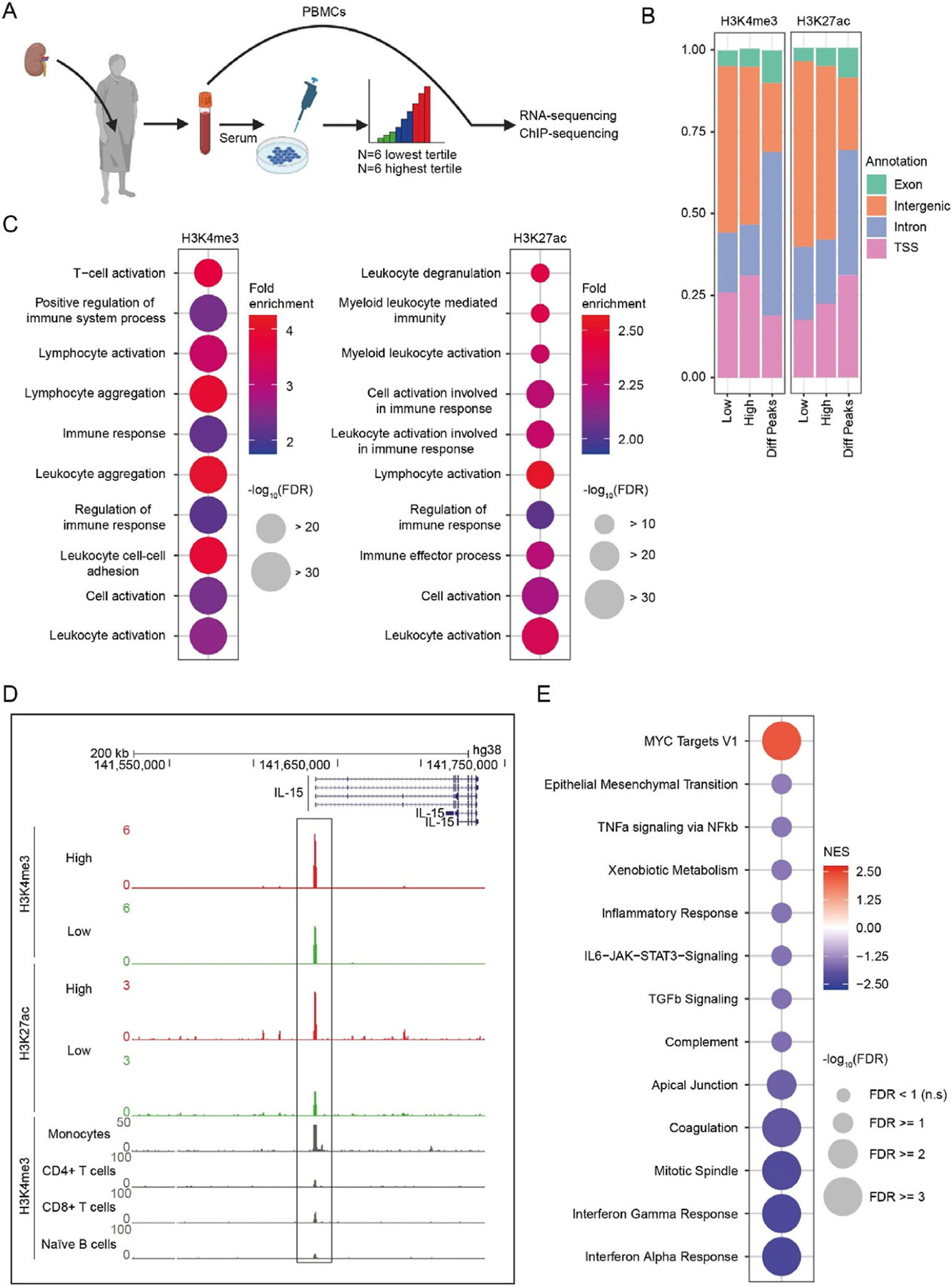
Transcriptional and epigenetic profiles of kidney transplant recipient’s circulating leukocytes. (A) Schematic representation of the method of selection of PBMCs of kidney transplant patients for RNA- and ChIP-sequencing. (B) Genomic annotations of H3K4me3 and H3K27ac peaks in the mean of 6 patients from the lowest tertile and 6 patients from the highest tertile compared to the differential peaks (FDR < 0.05, Diff Peaks panel). (C) Top 10 Gene Ontology (GO) Biological Processes associated with genomic regions showing altered H3K4me3 and H3K27ac in PBMCs of 6 patients from the lowest tertile and 6 patients from the highest tertile of posttransplant serum-induced trained immunity, determined using the Genomic Regions Enrichment of Annotations Tool (FC > 2, FDR < 0.05). (D) H3K4me3 and H3K27ac signal at *IL15* gene as visualized in the UCSC genome browser. High represents patients from the highest tertile (red), Low represents patients from the lowest tertile (green). In gray, the expression of *IL15* is displayed in monocytes, CD4+ T cells, CD8+ T cells, and naïve B cells. (E) Significantly altered gene sets of the Hallmark database in PBMCs of 6 patients from the lowest tertile and 6 patients from the highest tertile of posttransplant serum-induced trained immunity. ChIP, chromatin immunoprecipitation; FC, fold change; FDR, false discovery rate; H3K27ac, histone 3 lysine 27 acetylation; H3K4me3, histone 3 lysine 4 trimethylation; *IL15*, interleukin 15; MYC, myelocytomatosis oncogene; NES, normalized enrichment score; PBMC, peripheral blood mononuclear cell; TNF, tumor necrosis factor; TSS, transcription start site; ns, not significant; UCSC, University of California, Santa Cruz.

We investigated whether the trained immunity-regulating properties of patient serum correlated with changes in the epigenetic and transcriptional profiles of circulating leukocytes from kidney transplant recipients. For this purpose, we used PBMCs obtained 1 week after transplantation from 12 patients. Six were from the lowest and 6 from the highest tertile of responses in the trained immunity assay with posttransplant serum (Fig. 5A). Patient characteristics are described in Supplementary Table 9. We performed whole-genome RNA-seq and whole-genome assessment of H3K4me3 and H3K27ac by ChIP-seq.

ChIP-seq analysis showed a global increase in the number of H3K4me3 (1304 genes increased and 8 genes decreased, log2 fold change > 2, *P* <.05) and H3K27ac (1853 genes increased, 131 genes decreased, log2 fold change > 2, *P* <.05) peaks in the PBMCs from patients in the lowest tertile, with most differential H3K4me3 and H3K27ac peaks located at the introns and transcription start sites (Fig. 5B, Supplementary Fig. S23). Pathway analysis of the genes closest to the differentially regulated H3K4me3 and H3K27ac peaks, performed using the Genomic Regions of Annotations Tool, revealed differentially regulated pathways associated with leukocyte activation (Fig. 5C). We investigated signature genes of monocytes and found more H3K4me3 and H3K27ac at *IL15* (Fig. 5D). We also looked at H3K4me3 and H3K27ac marks on signature genes of T cell differentiation. We found more H3K4me3 and H3K27ac at *GATA3* (Supplementary Fig. S24, Supplementary Table 10).

We then used our RNA-seq data to perform a gene set enrichment analysis. Using the Molecular Signatures Database Hallmark gene set collection, we found increased expression of genes regulated by the myelocytomatosis oncogene transcription factor in PBMCs from patients in the lowest tertile. Furthermore, we found a marked decrease in transcription of genes related to IFN signaling, IL-6-JAK-STAT3 signaling, and TNF signaling via NFκβ, pointing toward a less inflammatory transcriptional signature of PBMCs from the lowest tertile (Fig. 5E).

## 4. Discussion

This study demonstrates the crucial role of trained immunity in kidney transplantation. We show that the serum of patients 1 week after transplantation induces trained immunity in vitro to a much lesser degree compared to pretransplant serum. The immunosuppressive drugs patients receive after transplantation, particularly prednisolone and tacrolimus, likely contribute to mediating these effects, although we found IRI to have little effect on the trained immune response. We revealed that the suppression of trained immunity by serum obtained 1 week posttransplant is associated with changes in circulating leukocyte’s epigenetic and transcriptomic profiles and discovered that patients whose serum most strongly suppresses trained immunity rarely experience graft loss.

Trained immunity likely evolved under the evolutionary pressure of infectious diseases. However, this could have adverse effects in the context of organ transplantation. In an experimental study using a mouse model of heart transplantation, we previously demonstrated that trained immunity regulates acute rejection in organ transplantation.^27^ We showed that allograft acceptance was substantially promoted by inhibiting trained immunity with mTOR inhibiting nanobiologics with a high avidity for innate immune cells.^27^ This encouraging result raised the promise that by therapeutically suppressing trained immunity, graft survival can be improved, fulfilling a long-standing clinical need. However, substantial differences between the experimental mouse model and the human situation prevent us from translating our findings directly into the human context. In contrast to mice, human kidney transplant recipients do not have a naïve immune system before transplantation, and patients receive various immunosuppressive drugs after transplantation that can affect innate immune responses. Therefore, it is crucial to understand the relevance of trained immunity in the clinical context of kidney transplantation.

Our study revealed that the degree to which serum obtained 1 week after kidney transplantation suppresses trained immunity is an independent predictor of kidney allograft survival. The difference between trained immunity tertiles was substantial, noting that in patients whose serum most potently suppressed trained immunity, virtually no graft loss occurred. Moreover, this relationship between trained immunity and graft survival was independent of other known clinical factors associated with graft survival.

The question is how trained immunity can influence graft survival so strongly. One possible hypothesis is that trained immunity promotes acute rejection. However, we did not find a relationship between trained immunity and the incidence of BPAR in the first 2 years after kidney transplantation. An alternative explanation could be that trained immunity is involved in developing persistent low-grade inflammation that promotes chronic fibrosis and chronic rejection of the allograft.^17,28^ Interstitial fibrosis of kidney transplants starts as early as the first months after transplantation, and fibrosis 1 year after transplantation is associated with reduced graft survival.^16,29–34^ Early infiltration of macrophages in allogeneic kidney transplant biopsies is linked to the development of interstitial fibrosis, and macrophage infiltration in areas with interstitial fibrosis is a strong predictor of graft survival.^33,35–37^ It is conceivable that increased inflammatory activity driven by trained immunity could accelerate graft fibrosis.^38^ Trained immunity could also fuel T cell alloimmunity and thereby propel chronic rejection. This should be examined by evaluating the role of trained immunity in driving graft fibrosis and chronic rejection using longitudinal kidney biopsies in future studies.

We investigated if sterile inflammation caused by IRI, which is inevitable in kidney transplantation, could cause the suppressive effect on trained immunity we observed in the experiments with posttransplant serum. In vitro, we examined a panel of DAMPs selected based on the receptors they stimulate. We observed dose-dependent responses, with stimuli engaging the IL-1 receptor and TLR2 and TLR4 receptors inducing trained immunity, whereas stimuli activating non-TLRs, particularly the Complement 1q and P2X7 receptors, suppressed it. These divergent effects corroborate the findings of a previous study. Neidhart et al^39^ also found that DAMPs can either induce or suppress trained immunity depending on the type of DAMP and their concentration. With such a variable effect of DAMPs on trained immunity, the question is what the net effect is of the mix of DAMPs released into the circulation during IRI. In our IRI mouse model, we found no evidence of trained immunity induction by IRI in the spleen and bone marrow. In addition, in kidney transplant recipients, we found no association between the trained immunity-inducing properties of patient sera and the occurrence of delayed graft function.

We found that immunosuppressive drugs, used as standard of care in kidney transplantation, modulate trained immunity. In particular, we found prednisolone and tacrolimus to have a potent suppressive effect on the trained immune response. For prednisolone, this effect has not been described previously. As for tacrolimus, there was a previous description of a suppressive effect on trained immunity by Fanucchi et al.^40^ They showed that β-glucan-trained monocytes increase the expression of upstream master long noncoding RNA of the inflammatory chemokine locus and other immune gene priming long noncoding RNAs. When monocytes were pretreated with tacrolimus before exposure to β-glucan, upregulation of upstream master long noncoding RNA of the inflammatory chemokine locus and other immune gene priming long noncoding RNAs relative to baseline levels was prevented, providing an explanatory mechanism for how tacrolimus can block the induction of trained immunity.^40^ Our finding that prednisolone and tacrolimus can inhibit trained immunity could explain, at least in part, why patient’s posttransplant serum suppresses trained immunity compared to their pretransplant serum. However, we do not exclude that other as yet unknown mechanisms, such as the interaction between immunosuppressive drugs and DAMPs, may also contribute to the suppressive effect of posttransplant serum on trained immunity.

We found that trained immunity suppression by patient serum is associated with a genome-wide increase in histone methylation and acetylation (H3K4me3 and H3K27ac) in circulating leukocytes, indicating a more open chromatin configuration and a more active gene transcription state. Interestingly, previous studies have shown that increasing histone acetylation, through inhibition of histone deacetylase, has anti-inflammatory properties and promotes the development of Tregs.^41,42^ Experimental bone marrow, heart, and islet transplantation models revealed that histone deacetylase inhibition promotes tolerance to the allograft.^41,42^ Looking at genes predominantly expressed by monocytes, we found that trained immunity suppression is associated with lower H3K4me3 and H3K27ac at *IL15*. IL-15 induces potent proliferative responses of activated and memory T and natural killer cells.^43^ Looking at transcription factors that regulate T cell differentiation, we observed that trained immunity suppression is associated with an increase in H3K4me3 and H3K27ac at *GATA3*. This transcription factor plays a crucial role in immune tolerance by regulating Treg function.^44^ An exciting finding from our RNA-seq data is that in patients whose serum suppressed trained immunity, there was an apparent decrease in the expression of inflammation-related genes, including genes involved in IFN signaling, IL-6-JAK-STAT3 signaling, and TNF signaling via NFκβ. We cannot unravel from our data whether this suppressed inflammatory profile in circulating leukocytes is caused by inhibition of trained immunity, the result of coincidental effects of immunosuppressive drugs, or attributable to the pre-existing immunologic profiles of these patients. We will disentangle how trained immunity and systemic immune responses interact in future studies.

Our study has limitations. Anti-CD20 therapy is not used as induction therapy in renal transplantation, unlike the study from which we obtained our samples. Future research will investigate patients receiving basiliximab or antithymocyte globulin induction. In vitro, rituximab may adhere to the cell surface and not be removed during washing, potentially affecting PBMCs. Additionally, serum and stimulus were only with PBMCs for the first 24 hours, differing from in vivo conditions.

Collectively, our findings indicate that trained immunity plays a role in kidney allograft survival. We found that posttransplant patient serum suppresses the trained immune response, at least partly mediated by immunosuppressive drugs. We showed that circulating leukocytes from patients whose serum most strongly inhibits the trained immune response have a more suppressed inflammatory profile. In these patients, graft loss rarely occurs. Combined, our data provide new pathophysiological insights into the role of innate immune cells in kidney transplant survival and identify trained immunity as a potential therapeutic target for improving graft survival.

## Supplementary Material

Multimedia component 1

Multimedia component 2

## Data Availability

The data that support the findings of this study are available on request from the corresponding author.

## Abbreviations:

BPAR: biopsy-proven acute rejection
ChIP-seq: chromatin immunoprecipitation sequencing
CI: confidence interval
DAMP: danger-associated molecular pattern
HKCA: heat-killed *Candida albicans*
H3K27ac: histone 3 lysine 27 acetylation
H3K4me3: histone 3 lysine 4 trimethylation
IFN: interferon
IL: interleukin
IRI: ischemia-reperfusion injury
LPS: lipopolysaccharide
PBMC: peripheral blood mononuclear cell
RNA-seq: ribonucleic acid sequencing
ROC: receiver operating characteristic
SD: standard deviation
TNF: tumor necrosis factor
