# Supplementary material for: Trained immunity suppression determines kidney allograft survival
Source: Am J Transplant. Author manuscript; Available in PMC 2025 Nov 1. (PMC11789421; doi:10.1016/j.ajt.2024.08.006)
Supplement: Multimedia component 1 [file NIHMS2046486-supplement-Multimedia_component_1.docx]

| **Supplementary Table 1. Demographics and characteristics of transplant patients and donors** |
| --- |
| \| Characteristics \| N=96 \| \| --- \| --- \| \| **Recipients**  Age, years  Mean  Male, no. (%)  Cold ischemia time, hours  Mean  BMI  Mean  Comorbidities, no. (%)  Diabetes  Hypertension  Follow-up, years  Mean  Pre-emptive transplant, no. (%)  Etiology of ESRD, no. (%)  Diabetic nephropathy  Glomerulonephritis/ glomerulopathy  Urologic  Polycystic kidney disease  Hypertension/vascular  Other  Uncertain/unknown  Donor specific antibodies pre-transplantation, no. (%)  Negative  Positive  Unknown  Rituximab use, no. (%)  **Donors**  Age, years  Mean  Deceased, no (%) \| 52.4 (40.6 – 60.1)  50.4  67 (69.8%)  3.0 (2.3 – 16.5)  9.5  24.7 (22.3 – 27.3)  24.8  8 (8.3%)**  86 (89.6%)**  7.7 (5.2 – 8.6)  6.8  22 (22.9%)  6 (6.3%)  23 (24%)  9 (9.4%)  22 (22.9%)  12 (12.5%)  12 (12.5%)  12 (12.5%)  87 (90.6%)  2 (2.1%)  7 (7.3%)  43 (44.8%)  55.5 (48.3 – 62.0)  55.1  44 (45.8%) \| |
| Data is presented as the median (interquartile range) or number (no.) and percentage (%),* represents missing data of one person. ESRD = End-stage renal disease. |

| **Supplementary Table 2. Results of multivariate linear regression analysis with backward elimination of clinical parameters and IL-6 and TNF response of pre-transplant serum** |
| --- |
| \| Characteristics \| B \| 95% Confidence  Interval \| p-value \| \| --- \| --- \| --- \| --- \| \| **IL-6**  Age  BMI  Pre-transplant serum level of IL-6  **TNF**  Etiology of ESRD \| 0.013  0.050  0.000  -0.100 \| 0.003 – 0.023  0.004 – 0.096  0.000 – 0.000  -0.169 – -0.032 \| 0.015  0.034  0.046  0.004 \| |
| ESRD = End-stage renal disease.  Parameters included; age, gender, hypertension, diabetes, BMI, etiology of ESRD, preemptive transplant, pre-transplant serum level of IL-6, pre-transplant serum level of TNF |

| **Supplementary Table 3. Demographics and characteristics of transplant patients divided into tertiles according to the IL-6 response of post-transplantation serum-induced trained immunity** |
| --- |
| \| Characteristics \| N=32 (L) \| N=32 (M) \| N=32 (H) \| \| --- \| --- \| --- \| --- \| \| **Recipients**  Age, years  Mean  Male, no. (%)  Cold ischemia time, hours  Mean  BMI  Mean  Comorbidities, no. (%)  Diabetes  Hypertension  Follow-up, years  Mean  Pre-emptive transplant, no. (%)  Etiology of ESRD, no. (%)  Diabetic nephropathy  Glomerulonephritis/ glomerulopathy  Urologic  Polycystic kidney disease  Hypertension/vascular  Other  Uncertain/unknown  Donor specific antibodies pre-transplantation, no. (%)  Negative  Positive  Unknown  Rituximab use, no. (%)  **Donors**  Age, years  Mean  Deceased, no (%) \| 54.1 (45.3 – 60.0)  51.7  21 (65.6%)  5.8 (2.4 – 16.9)  9.6  25.2 (22.7 – 27.3)  25.2  2 (6.3%)  30 (93.8%)  7.1 (5.0 – 8.1)  6.3  6 (18.8%)  2 (6.3%)  10 (31.3%)  2 (6.3%)  11 (34.4%)  2 (6.3%)  2 (6.3%)  3 (9.4%)  28 (87.5%)  1 (3.1%)  3 (9.4%)  15 (46.9%)  53.0 (46.0 – 59.0)  52.9  16 (50.0%) \| 51.6 (39.8 – 61.5)  50.9  26 (81.3%)  2.9 (2.4 – 14.5)  8.7  24.7 (22.8 – 27.5)  25.1  5 (15.6%)*  27 (84.4%)*  8.2 (6.0 – 9.0)  7.3  9 (28.1%)  4 (12.5%)  6 (18.8%)  4 (12.5%)  5 (15.6%)  6 (18.8%)  4 (12.5%)  3 (9.4%)  30 (93.8%)  0 (0%)  2 (6.2%)  15 (46.9%)  56.5 (48.0 – 63.0)  56.0  14 (43.8%) \| 50.8 (36.3 – 59.9)  48.5  20 (62.5%)  2.9 (2.2 – 19.3)  10.3  23.8 (21.4 – 27.1)  24.2  1 (3.1%)*  29 (90.6%)*  7.9 (5.1 – 8.7)  6.7  7 (21.9%)  0 (0%)  7 (21.9%)  3 (9.4%)  6 (18.8%)  4 (12.5%)  6 (18.8%)  6 (18.8%)  29 (90.6%)  1 (3.1%)  2 (6.3%)  13 (40.6%)  56.5 (52.3 – 62.8)  56.3  14 (43.8%) \| |
| Data is presented as the median (interquartile range) or number (no.) and percentage (%),* represents missing data of one person. L = patients from the lowest tertitle of IL-6 post-transplantation serum-induced trained immunity, M = patients from the middle tertitle of IL-6 post-transplantation serum-induced trained immunity H = patients from the highest tertile of IL-6 post-transplantation serum-induced trained immunity. ESRD = End-stage renal disease. |

| **Supplementary Table 4. Demographics and characteristics of transplant patients divided into tertiles according to the TNF response of post-transplantation serum-induced trained immunity** |
| --- |
| \| Characteristics \| N=32 (L) \| N=32 (M) \| N=32 (H) \| \| --- \| --- \| --- \| --- \| \| **Recipients**  Age, years  Mean  Male, no. (%)  Cold ischemia time, hours  Mean  BMI  Mean  Comorbidities, no. (%)  Diabetes  Hypertension  Follow-up, years  Mean  Pre-emptive transplant, no. (%)  Etiology of ESRD, no. (%)  Diabetic nephropathy  Glomerulonephritis/ glomerulopathy  Urologic  Polycystic kidney disease  Hypertension/vascular  Other  Uncertain/unknown  Donor specific antibodies pre-transplantation, no. (%)  Negative  Positive  Unknown  Rituximab use, no. (%)  **Donors**  Age, years  Mean  Deceased, no (%) \| 52.3 (44.2 – 58.9)  50.0  21 (65.6%)  2.6 (2.3 – 11.5)  6.0  24.9 (23.6 – 27.5)  25.6  5 (15.6%)*  28 (87.5%)*  6.9 (5.0 – 8.5)  6.3  8 (25.0%)  4 (12.5%)  4 (12.5%)  1 (3.1%)  10 (31.3%)  5 (15.6%)  4 (12.5%)  4 (12.5%)  28 (87.5%)  1 (3.1%)  3 (9.4%)  13 (40.6%)  51.5 (46.2 – 57.0)  51.7  9 (28.1%) \| 50.8 (36.0 – 62.6)  50.3  24 (75.0%)  10.5 (2.4 – 17.6)  10.8  24.5 (22.2 – 27.0)  24.6  1 (3.1%)  29 (90.6%)  8.2 (7.3 – 9.1)  8.0  8 (25.0%)  1 (3.1%)  12 (37.5%)  5 (15.6%)  4 (12.5%)  3 (9.4%)  5 (15.6%)  2 (6.3%)  29 (90.6%)  1 (3.1%)  2 (6.3%)  17 (53.1%)  60.5 (52.3 – 63.0)  58.0  17 (53.1%) \| 53.0 (46.7 – 59.0)  50.8  22 (68.8%)  11.5 (2.2 – 20.3)  11.9  23.9 (21.4 – 27.4)  24.2  2 (6.3%)*  29 (90.6%)*  7.2 (3.8 – 8.2)  6.1  6 (18.8%)  1 (3.1%)  7 (21.9%)  3 (9.4%)  8 (25%)  4 (12.5%)  3 (9.4%)  6 (18.8%)  30 (93.8%)  0 (0%)  2 (6.3%)  13 (40.6%)  55.0 (49.5 – 62.0)  55.5  18 (56.3%) \| |
| Data is presented as the median (interquartile range) or number (no.) and percentage (%),* represents a missing person. L = patients from the lowest tertitle of TNF post-transplantation serum-induced trained immunity, M = patients from the middle tertitle of TNF post-transplantation serum-induced trained immunity H = patients from the highest tertile of TNF post-transplantation serum-induced trained immunity. ESRD = End-stage renal disease. |

| **Supplementary Table 5. Demographics and characteristics of transplant patients divided into tertiles according to the IL-6 response of pre-transplantation serum-induced trained immunity** |
| --- |
| \| Characteristics \| N=32 (L) \| N=32 (M) \| N=32 (H) \| \| --- \| --- \| --- \| --- \| \| **Recipients**  Age, years  Mean  Male, no. (%)  Cold ischemia time, hours  Mean  BMI  Mean  Comorbidities, no. (%)  Diabetes  Hypertension  Follow-up, years  Mean  Pre-emptive transplant, no. (%)  Etiology of ESRD, no. (%)  Diabetic nephropathy  Glomerulonephritis/ glomerulopathy  Urologic  Polycystic kidney disease  Hypertension/vascular  Other  Uncertain/unknown  Donor specific antibodies pre-transplantation, no. (%)  Negative  Positive  Unknown  Rituximab use, no. (%)  **Donors**  Age, years  Mean  Deceased, no (%) \| 50.5 (35.8 – 59.6)  47.8  23 (71.9%)  2.9 (2.4 – 17.9)  9.4  23.9 (21.9 – 26.6)  24.3  3 (9.4%)*  29 (90.6%)*  7.3 (5.1 – 8.5)  6.8  9 (28.1%)  3 (9.4%)  4 (12.5%)  5 (15.6%)  6 (18.8%)  3 (9.4%)  6 (18.8%)  5 (15.6%)  29 (90.6%)  0 (0.0%)  3 (9.4%)  21 (65.6%)  57.5 (49.3 – 62.0)  56.8  13 (40.6%) \| 49.8 (33.8 – 58.5)  46.4  19 (59.4%)  2.5 (2.2 – 14.2)  8.6  24.1 (20.7 – 27.1)  24.0  2 (6.3%)*  27 (84.4%)*  7.6 (4.1 – 8.6)  6.3  7 (21.9%)  1 (3.1%)  7 (21.9%)  3 (9.4%)  8 (25.0%)  6 (18.8%)  3 (9.4%)  4 (12.5%)  30 (93.8%)  0 (0.0%)  2 (6.3%)  11 (34.4%)  52.5 (45.5 – 58.0)  51.3  13 (40.6%) \| 57.5 (50.4 – 61.5)  56.8  25 (78.1%)  12.6 (2.3 – 16.8)  10.6  26.1 (24.4 – 27.6)  26.2  3 (9.4%)  30 (93.8%)  7.9 (6.5 – 8.6)  7.2  6 (18.8%)  2 (6.3%)  12 (37.5%)  1 (3.1%)  8 (25.0%)  3 (9.4%)  3 (9.4%)  3 (9.4%)  28 (87.5%)  2 (6.3%)  2 (6.3%)  11 (34.4%)  58.0 (48.8 – 64.0)  57.1  18 (56.3%) \| |
| Data is presented as the median (interquartile range) or number (no.) and percentage (%),* represents missing data of one person. L = patients from the lowest tertitle of IL-6 pre-transplantation serum-induced trained immunity, M = patients from the middle tertitle of IL-6 pre-transplantation serum-induced trained immunity H = patients from the highest tertile of IL-6 post-transplantation serum-induced trained immunity. ESRD = End-stage renal disease. |

| **Supplementary Table 6. Demographics and characteristics of transplant patients divided into tertiles according to the TNF response of pre-transplantation serum-induced trained immunity** |
| --- |
| \| Characteristics \| N=32 (L) \| N=32 (M) \| N=32 (H) \| \| --- \| --- \| --- \| --- \| \| **Recipients**  Age, years  Mean  Male, no. (%)  Cold ischemia time, hours  Mean  BMI  Mean  Comorbidities, no. (%)  Diabetes  Hypertension  Follow-up, years  Mean  Pre-emptive transplant, no. (%)  Etiology of ESRD, no. (%)  Diabetic nephropathy  Glomerulonephritis/ glomerulopathy  Urologic  Polycystic kidney disease  Hypertension/vascular  Other  Uncertain/unknown  Donor specific antibodies pre-transplantation, no. (%)  Negative  Positive  Unknown  Rituximab use, no. (%)  **Donors**  Age, years  Mean  Deceased, no (%) \| 50.2 (40.7 – 59.0)  49.4  22 (68.8%)  2.5 (3.5 – 16.3)  9.4  24.8 (21.9 – 27.9)  25.0  2 (6.3%)*  28 (87.5%)*  7.0 (5.3 – 8.6)  6.8  5 (15.6%)  2 (6.3%)  7 (21.9%)  3 (9.4%)  6 (6.3%)  3 (9.4%)  6 (18.8%)  5 (15.6%)  28 (87.5%)  1 (3.1%)  3 (9.4%)  17 (53.1%)  56.0 (50.3 – 62.8)  56.1  15 (46.9%) \| 51.0 (32.1 – 59.8)  47.3  21 (65.6%)  2.8 (2.2 – 17.2)  9.7  23.5 (20.6 – 25.0)  23.5  3 (9.4%)*  28 (87.5%)*  7.4 (5.0 – 8.3)  6.5  10 (31.3%)  2 (6.3%)  5 (15.6%)  2 (6.3%)  8 (25.0%)  7 (21.9%)  4 (12.5%)  4 (12.5%)  30 (93.8%)  0 (0.0%)  2 (6.3%)  14 (43.8%)  54.5 (46.0 – 62.0)  54.3  14 (43.8%) \| 53.3 (47.8 – 61.1)  54.4  24 (75.0%)  2.7 (2.3 – 16.8)  9.5  26.1 (24.0 – 27.5)  26.0  3 (9.4%)  30 (93.8%)  7.9 (5.6 – 8.5)  7.1  7 (21.9%)  2 (6.3%)  11 (34.4%)  4 (12.5%)  8 (25.0%)  2 (6.3%)  2 (6.3%)  3 (9.4%)  29 (90.6%)  1 (3.1%)  2 (6.3%)  12 (37.5%)  55.5 (47.3 – 61.0)  54.8  15 (46.9%) \| |
| Data is presented as the median (interquartile range) or number (no.) and percentage (%),* represents missing data of one person. L = patients from the lowest tertitle of TNF pre-transplantation serum-induced trained immunity, M = patients from the middle tertitle of TNF pre-transplantation serum-induced trained immunity H = patients from the highest tertile of TNF pre-transplantation serum-induced trained immunity. ESRD = End-stage renal disease. |

| **Supplementary Table 7. Cox proportional-hazards analysis for the tertiles of the IL-6 response** |
| --- |
| \| Characteristics \| Exp (B) \| 95% Confidence  Interval \| p-value \| \| --- \| --- \| --- \| --- \| \| Age  Gender  BMI  Hypertension  Diabetes  Etiology of ESRD  Pre-emptive transplant  Placebo vs rituximab  Age donor  Type donor  HLA mismatch  Tertiles of IL-6 response \| 0.998  1.437  1.150  3.395  397506  1.116  261815  0.370  1.030  0.241  1.008  4.421 \| 0.936 – 1.065  0.289 – 7.146  0.935 – 1.415  0.715 – 16.117  0.000 – .  0.793 – 1.573  0.000 – 4.529^259^  0.074 – 1.849  0.946 – 1.121  0.046 – 1.255  0.516 – 1.971  1.515 – 12.901 \| 0.956  0.658  0.185  0.124  0.974  0.528  0.967  0.226  0.499  0.091  0.981  0.007 \| |
| ESRD = End-stage renal disease. |

| **Supplementary Table 8. Cox proportional-hazards analysis for the tertiles of the TNF response** |
| --- |
| \| Characteristics \| Exp (B) \| 95% Confidence  Interval \| p-value \| \| --- \| --- \| --- \| --- \| \| Age  Gender  BMI  Hypertension  Diabetes  Etiology of ESRD  Pre-emptive transplant  Placebo vs rituximab  Age donor  Type donor  HLA mismatch  Tertiles of TNF response \| 0.952  1.153  1.389  8.064  398100  1.496  269746  0.619  1.068  0.199  1.118  6.220 \| 0.885 – 1.023  0.289 – 4.595  1.086 – 1.776  1.860 – 34.971  0.000 – .  1.105 – 2.025  0.000 – 3.941^287^  0.181 – 2.116  0.974 – 1.171  0.033 – 1.206  0.541 – 2.308  1.889 – 20.485 \| 0.181  0.840  0.009  0.005  0.973  0.009  0.970  0.445  0.163  0.079  0.764  0.003 \| |
| ESRD = End-stage renal disease. |

| **Supplementary Table 9. Demographics and characteristics of transplant patients used for RNA-sequencing and ChIP-sequencing** |
| --- |
| \| Characteristics \| N=12 \| N=6 (L) \| N=6 (H) \| \| --- \| --- \| --- \| --- \| \| **Recipients**  Age, years  Mean  Male, no. (%)  Cold ischemia time, hours  Mean  BMI  Mean  Comorbidities, no. (%)  Diabetes  Hypertension  Follow-up, years  Mean  Pre-emptive transplant, no. (%)  Etiology of ESRD, no. (%)  Diabetic nephropathy  Glomerulonephritis/ glomerulopathy  Urologic  Polycystic kidney disease  Hypertension/vascular  Other  Uncertain/unknown  Donor specific antibodies pre-transplantation, no. (%)  Negative  Positive  Unknown  Rituximab use, no. (%)  **Donors**  Age, years  Mean  Deceased, no (%) \| 56.4 (47.1 – 64.8)  54.1  7 (58.3%)  7.9 (2.3 – 17.0)  9.6  24.6 (22.1 – 26.6)  24.8  1 (8.3%)*  11 (91.7%)*  6.6 (5.0 – 7.8)  6.2  0 (0.0%)  1 (8.3%)  4 (33.3%)  0 (0.0%)  2 (16.7%)  3 (25.0%)  1 (8.3%)  1 (8.3%)  11 (91.7%)  1 (8.3%)  0 (0.0%)  5 (41.7%)  57.5 (51.3 – 64.0)  56.0  6 (50.0%) \| 56.4 (41.5 – 62.6)  53.3  2 (33.3%)  7.9 (2.4 – 16.5)  9.0  25.7 (23.8 – 28.9)  26.2  1 (16.7%)  6 (100%)  6.9 (4.7 – 8.0)  6.5  0 (0.0%)  1 (16.7%)  3 (50.0%)  0 (0.0%)  2 (33.3%)  0 (0.0%)  0 (0.0%)  0 (0.0%)  5 (83.3%)  1 (16.7%)  0 (0.0%)  2 (33.3%)  58.5 (52.0 – 64.5)  56.8  3 (50.0%) \| 56.5 (46.7 – 67.9)  54.8  5 (83.3%)  9.8 (2.0 – 18.5)  10.2  22.9 (21.2 – 26.0)  23.3  0 (0.0%)*  5 (83.3%)*  5.7 (4.4 – 7.9)  5.9  0 (0.0%)  0 (0.0%)  1 (16.7%)  0 (0.0%)  0 (0.0%)  3 (50.0%  1 (16.7%)  1 (16.7%)  6 (100%)  0 (0.0%)  0 (0.0%)  3 (50%)  52.5 (48.0 – 66.0)  55.2  3 (50.0%) \| |
| Data is presented as the median (inter quartile range) or number (no.) and percentage (%),* represents missing data of one person. L = 6 patients from the lowest tertitle of TNF and IL-6 post-transplantation serum-induced trained immunity, H = 6 patients from the highest tertile of TNF and IL-6 post-transplantation serum-induced trained immunity. ESRD = End stage renal disease. |

| **Supplementary Table 10. Signature genes of T cell differentiation** |
| --- |
| \|  \| Log2 fold change \| Adjusted p-value \| \| --- \| --- \| --- \| \| **H3K4me3**  *T-bet* (*TBX21*)  *GATA3*  *FOXP3*  *RORγT* (*RORC*)  **H3K27ac**  *T-bet* (*TBX21*)  *GATA3*  *FOXP3*  *RORγT* (*RORC*) \| 1.49  2.60  -  1.45  2.29  3.33  -  1.60 \| 0.001  1.01*10^-5^  -  0.005  1.80*10^-4^  1.55*10^-6^  -  0.042 \| |

| 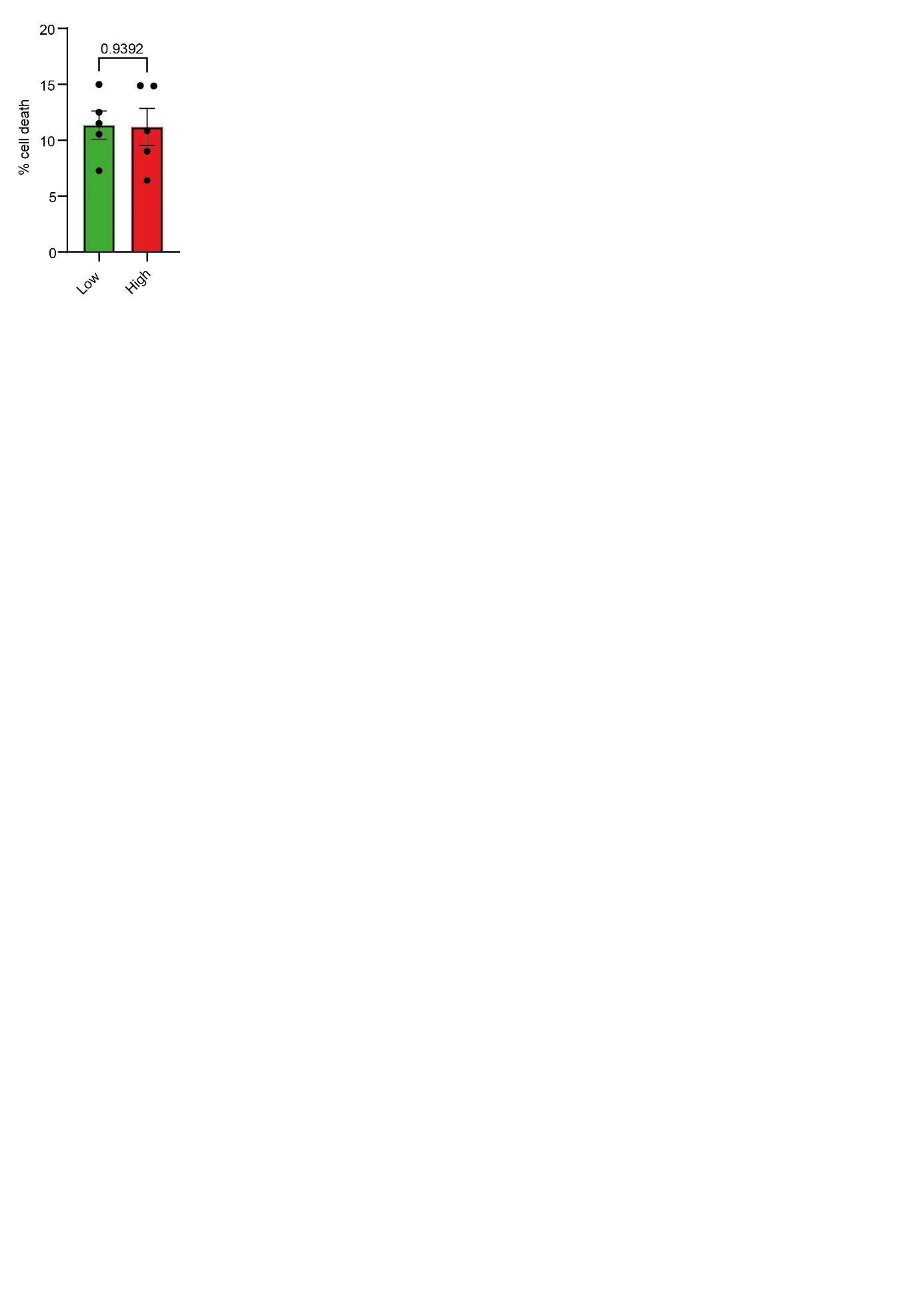 |
| --- |
| **Supplementary figure 1: Cell death after five-day resting period of PBMCs stimulated with post-transplant patient serum** Annexin/PI staining results of comparison of cell death after five-day resting period of PBMCs stimulated with post-transplant serum of five patients that induced the lowest IL-6 and TNF trained immunity response and serum of five patients that induced the highest IL-6 and TNF trained immunity response. |

| 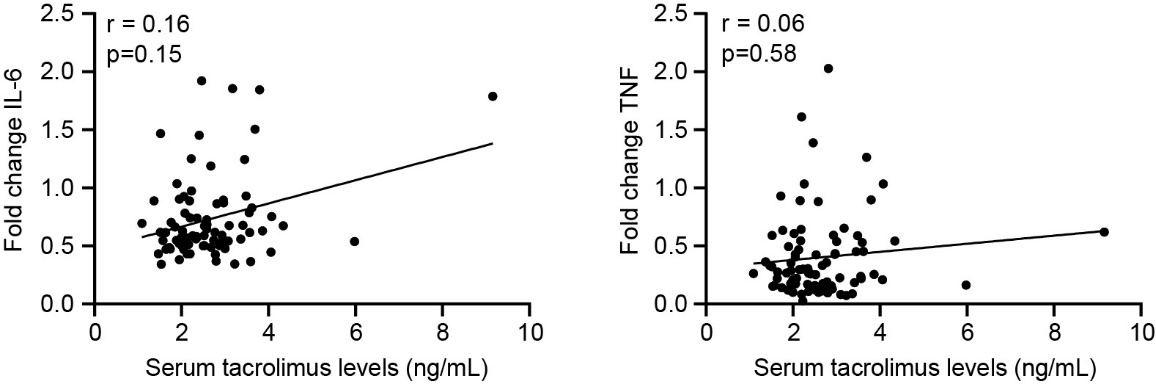 |
| --- |
| **Supplementary figure 2: Correlation between IL-6 and TNF levels measured in the supernatant after post-transplant serum trained immunity assay and their serum tacrolimus levels** Spearman’s rho correlation between IL-6 and TNF levels measured in the supernatant after post-transplant serum trained immunity assay and their serum tacrolimus levels. |

| 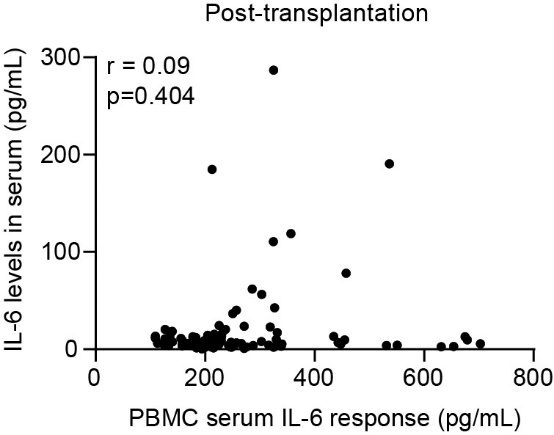 |
| --- |
| **Supplementary figure 3: No correlation between post-transplantation serum IL-6 levels and PBMC post-transplantation serum IL-6 response** Correlation between post-transplantation serum IL-6 levels and PBMC post-transplantation serum IL-6 response of 96 kidney transplant recipients. |

| 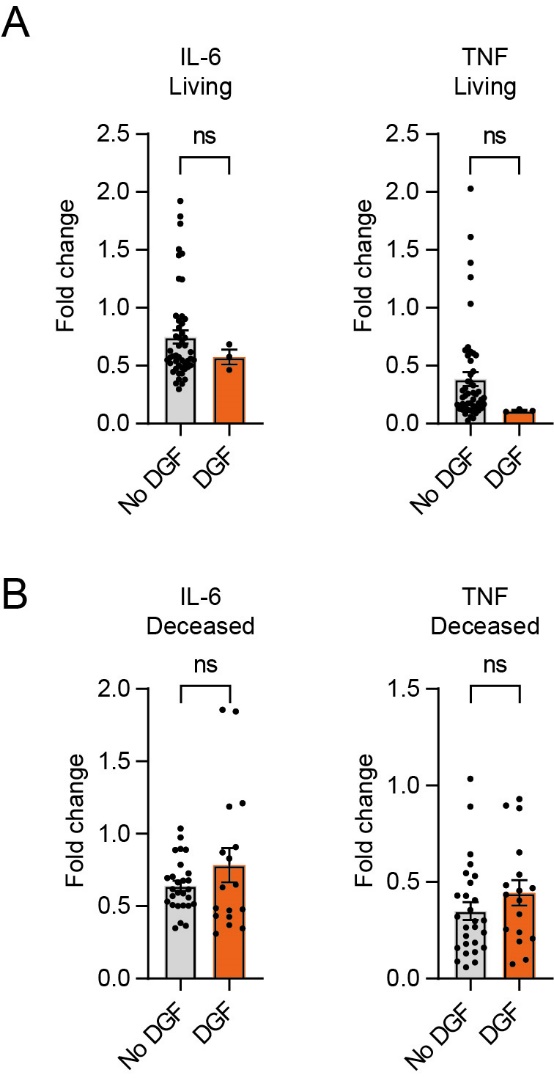 |
| --- |
| **Supplementary figure 4: No differences in IL-6 and TNF response between patients with delayed graft function compared to no delayed graft function A.B.** Comparison between IL-6 and TNF response in cells trained with post-transplant serum from patients with delayed graft function (DGF) compared to patients with no delayed graft function (no DGF) separated for patients who received a kidney from a living donor (A), and patients who received a kidney from a deceased donor (B). |

| **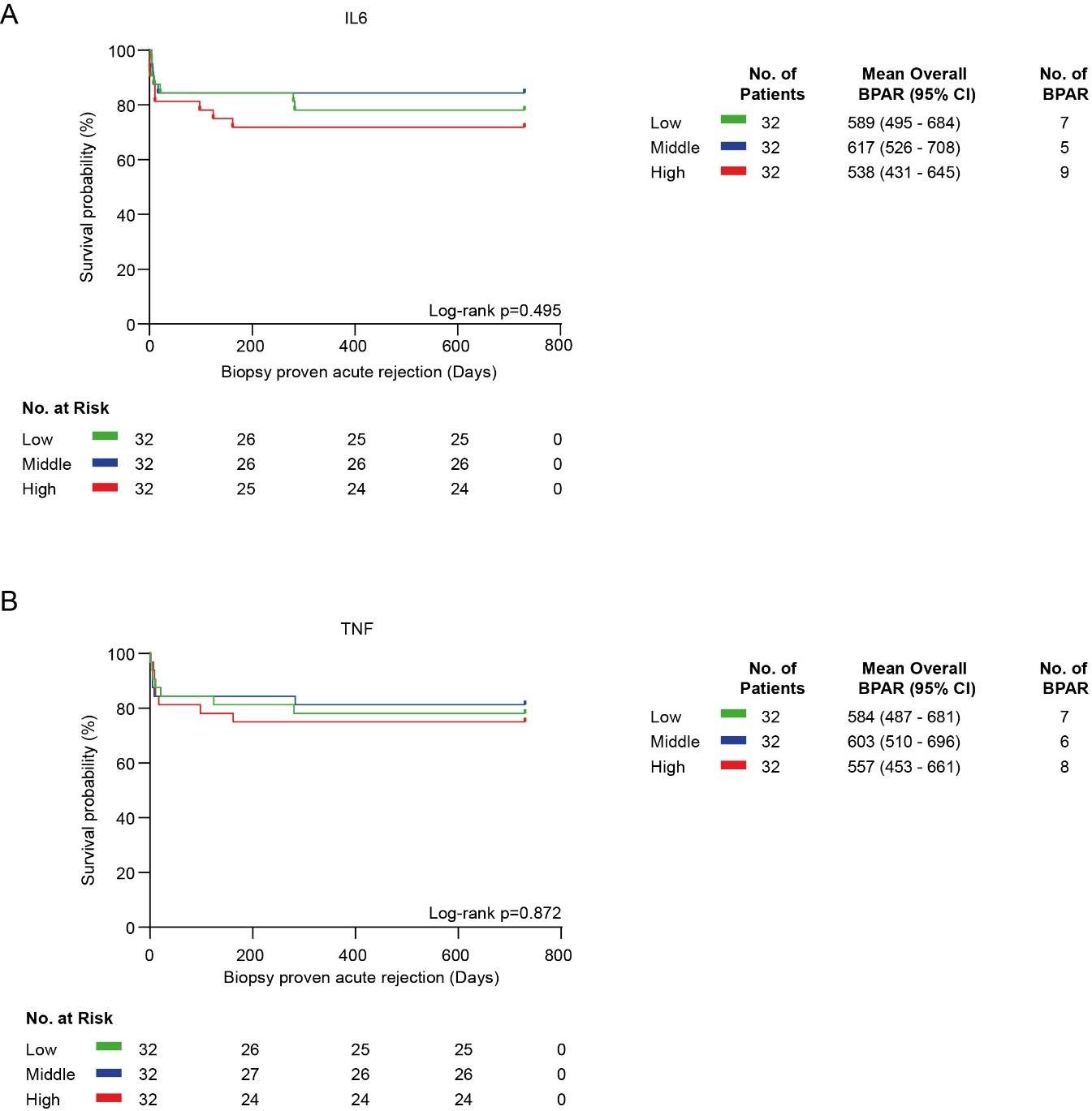** |
| --- |
| **Supplementary figure 5: Pre-transplant serum-induced cytokine responses are not associated with biopsy proven acute rejection A.B.** Kaplan-Meier survival analysis of biopsy proven acute rejection (BPAR) of the 3 tertiles of serum-induced trained immunity for the IL-6 and TNF response to LPS restimulation. p-values were calculated with a log-rank test. |

| **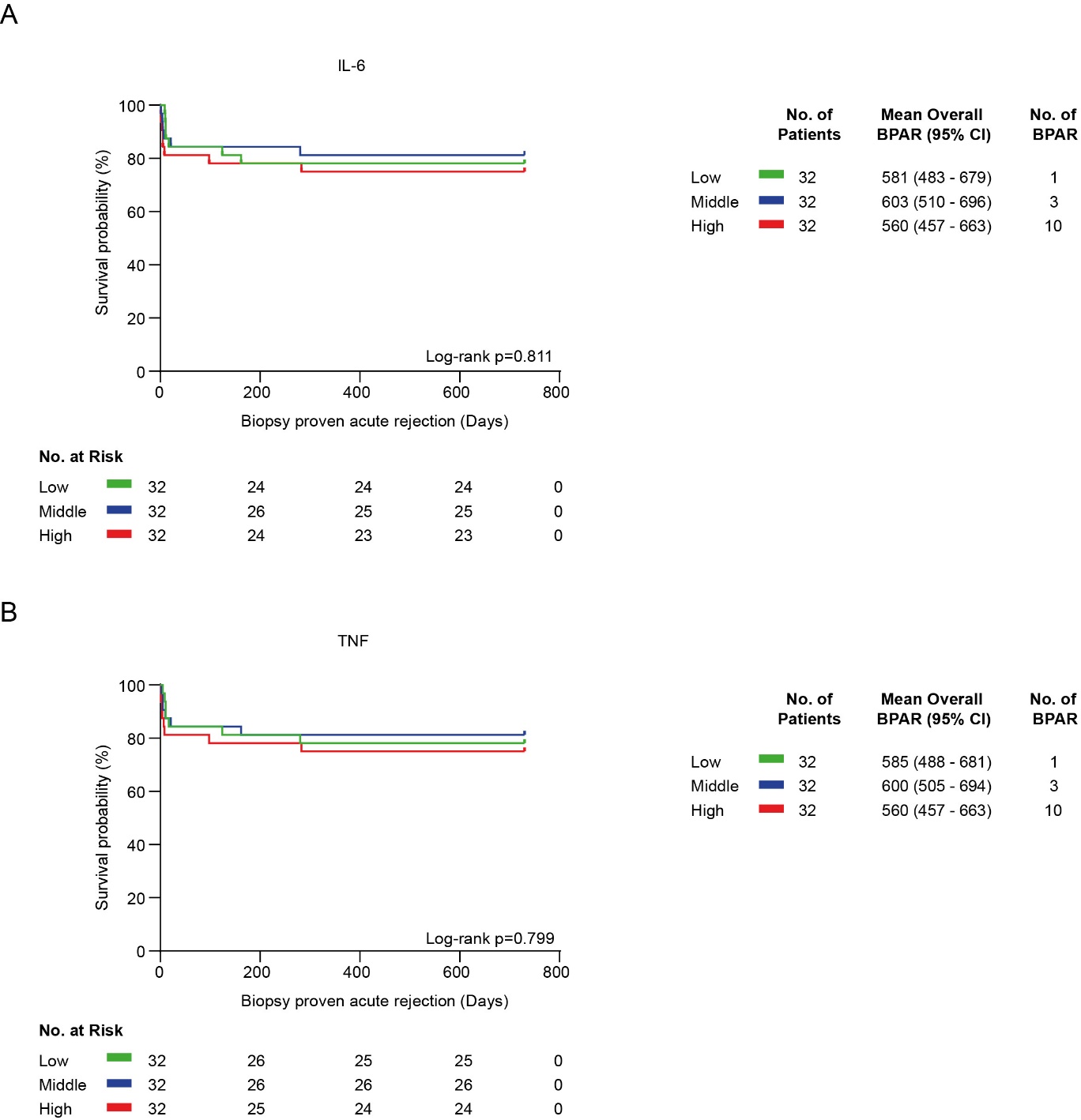** |
| --- |
| **Supplementary figure 6: Post-transplant serum-induced cytokine responses are not associated with biopsy proven acute rejection A.B.** Kaplan-Meier survival analysis of biopsy proven acute rejection (BPAR) of the 3 tertiles of serum-induced trained immunity for the IL-6 and TNF response to LPS restimulation. p-values were calculated with a log-rank test. |

| 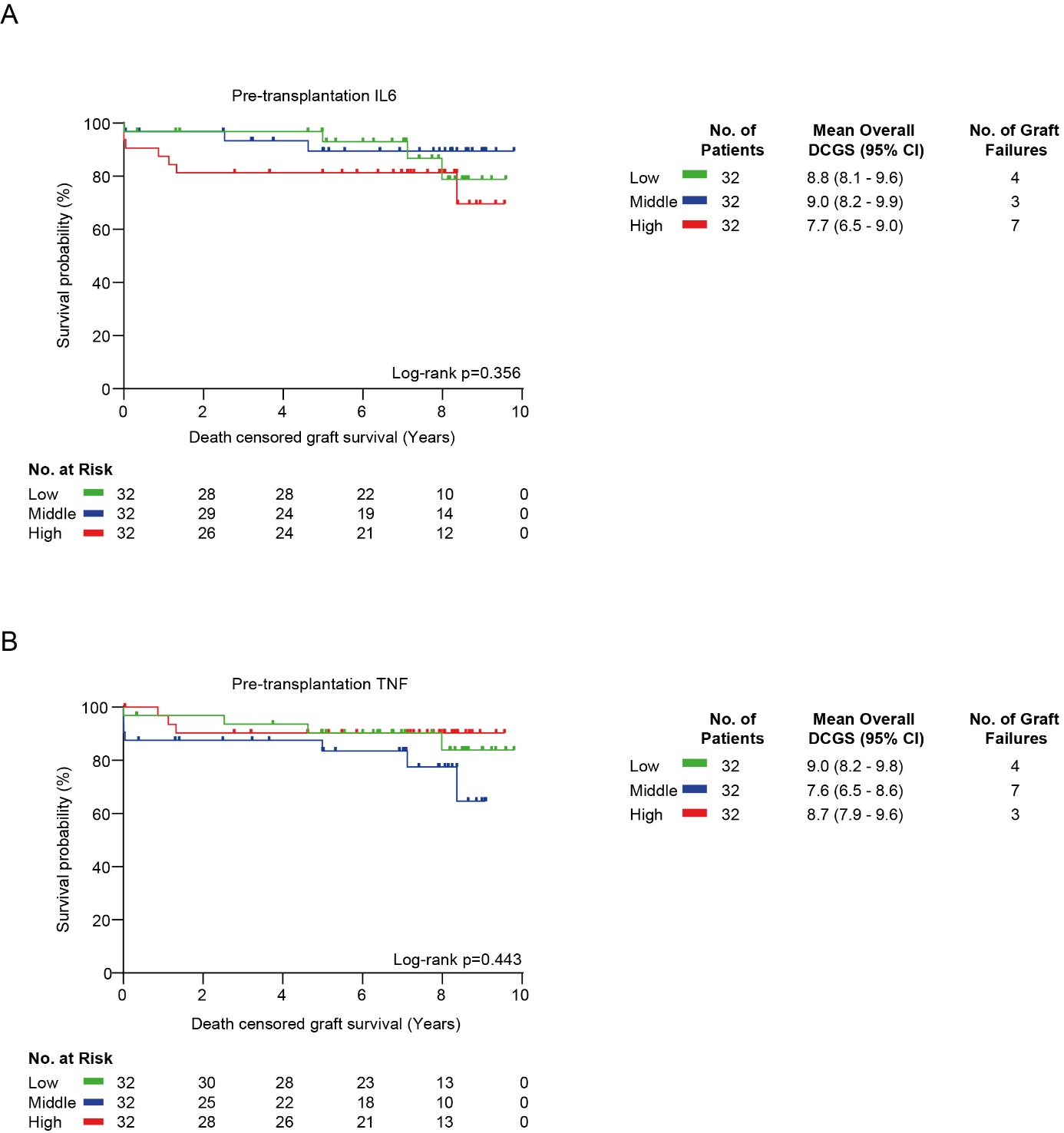 |
| --- |
| **Supplementary figure 7: Pre-transplant serum-induced cytokine responses are not associated with long-term graft survival A.B.** Kaplan-Meier survival analyses of death-censored graft survival of the tertiles of serum-induced trained immunity for the IL-6 and TNF responses to LPS restimulation. Data are expressed as survival probability (%) and death-censored graft survival (years). p-values were calculated with a Log-rank test. |

| 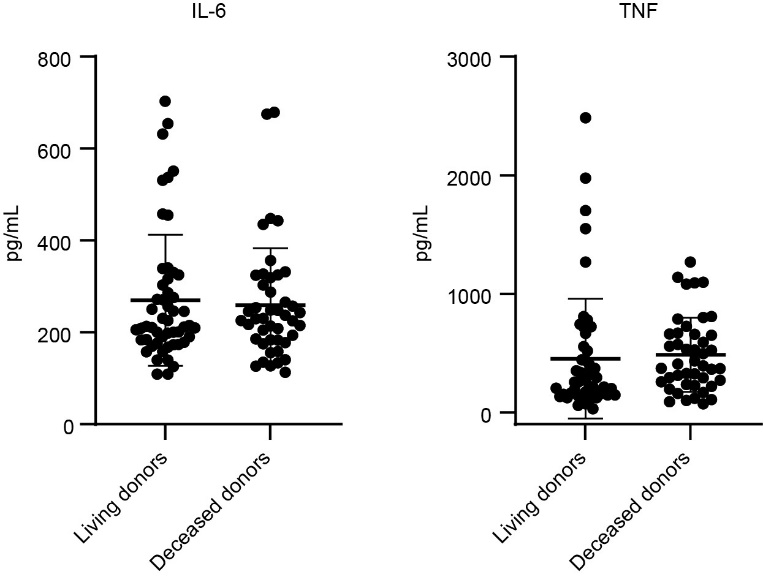 |
| --- |
| **Supplementary figure 8: No differences in the IL-6 and TNF trained immunity response of post-transplantation serum between patients receiving a kidney from a living donor versus from a deceased donor** Comparison between IL-6 and TNF trained immunity response of post-transplantation serum in patients receiving a kidney from a living donor versus patients receiving a kidney from a deceased donor. |

| 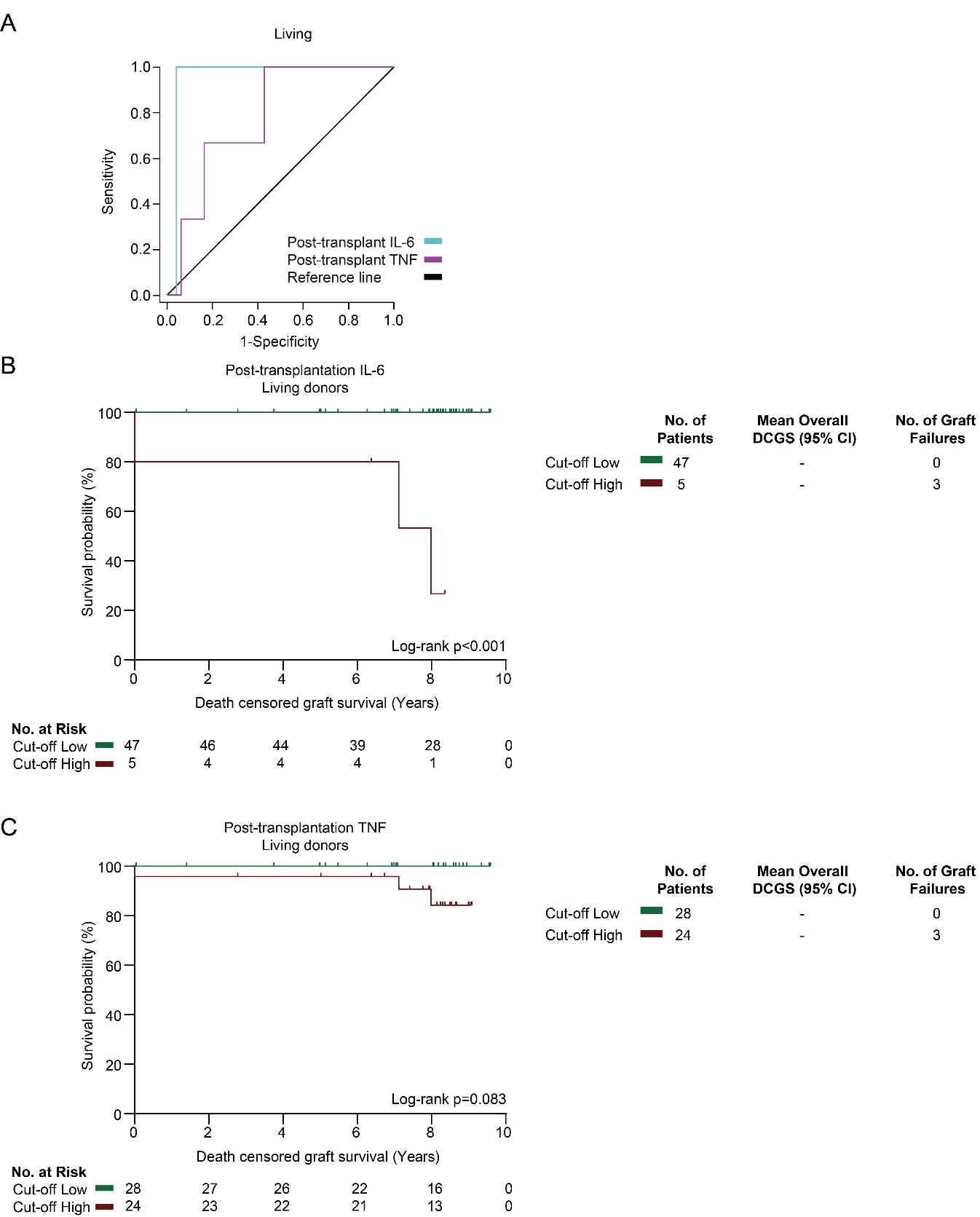 |
| --- |
| **Supplementary figure 9: Post-transplant serum-induced cytokine responses in patients who received a kidney from a living donor are associated with long-term graft survival A.** ROC curves for the IL-6 and TNF responses to LPS restimulation. AUC of 0.96 for the IL-6 response and 0.78 for the TNF response, with a post-transplant IL-6 response cut-off of 534 pg/mL, yielding a sensitivity of 100%, and a specificity of 95.9%, and a post-transplant TNF response cut-off of 285 pg/mL with a sensitivity of 100%, and a specificity of 57.1%. **B, C.** Kaplan-Meier survival analyses of death-censored graft survival of the low versus the high post-transplant serum-induced trained immunity for the IL-6 and TNF responses to LPS restimulation, based on the cut-offs determined by the ROC curves. Data are expressed as survival probability (%) and death-censored graft survival (years). p-values were calculated with a Log-rank test. |

| 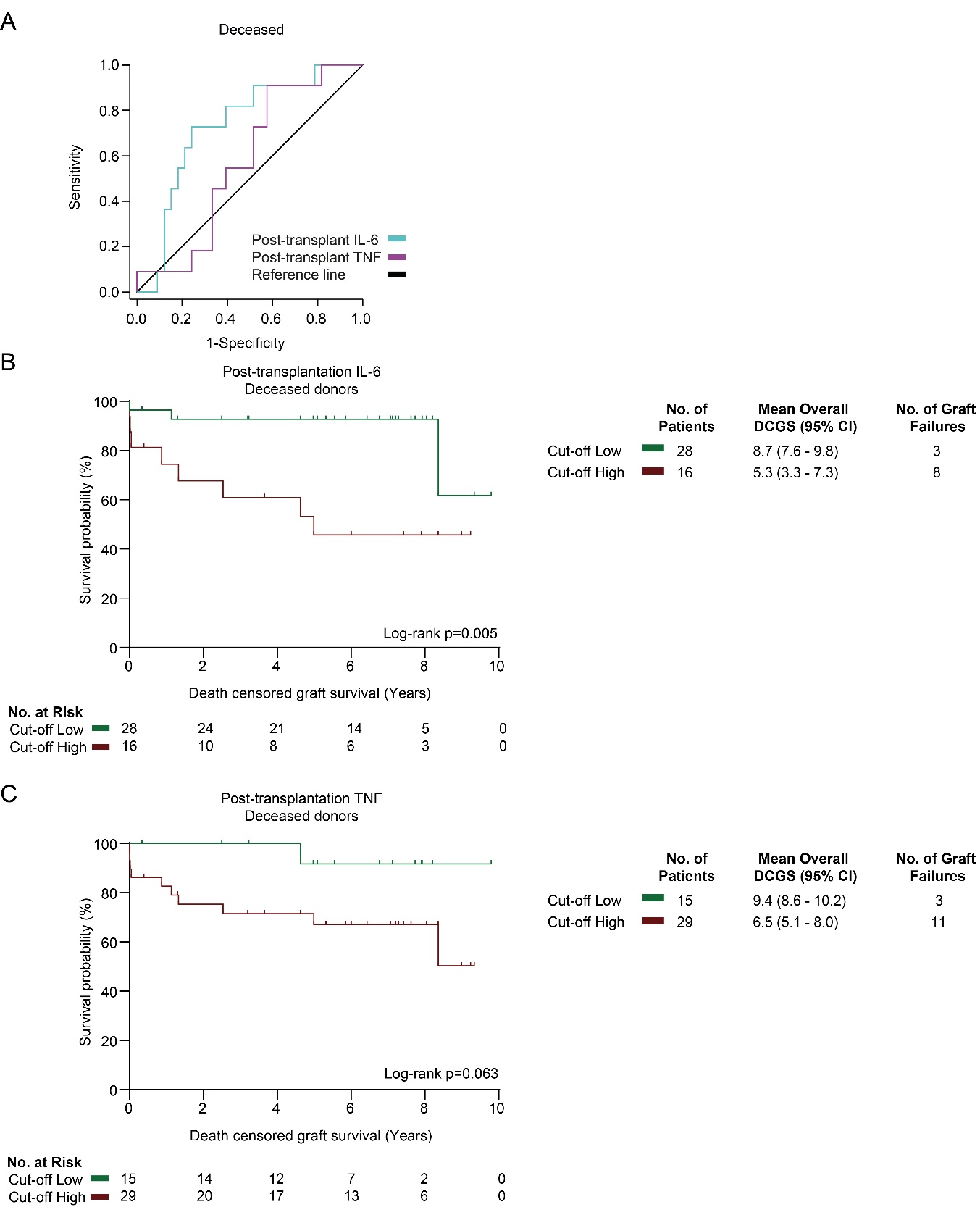 |
| --- |
| **Supplementary figure 10: Post-transplant serum-induced cytokine responses in patients who received a kidney from a deceased donor are associated with long-term graft survival A.** ROC curves for the IL-6 and TNF responses to LPS restimulation. AUC of 0.73 for the IL-6 response and 0.58 for the TNF response, with a post-transplant IL-6 response cut-off of 251 pg/mL, yielding a sensitivity of 72.7%, and a specificity of 75.8%, and a post-transplant TNF response the cut-off of 302 pg/mL with a sensitivity of 90.9% and a specificity of 42.4%. **B, C.** Kaplan-Meier survival analyses of death-censored graft survival of the low versus the high post-transplant serum-induced trained immunity for the IL-6 and TNF responses to LPS restimulation, based on the cut-offs determined by the ROC curves. Data are expressed as survival probability (%) and death-censored graft survival (years). p-values were calculated with a Log-rank test. |

| 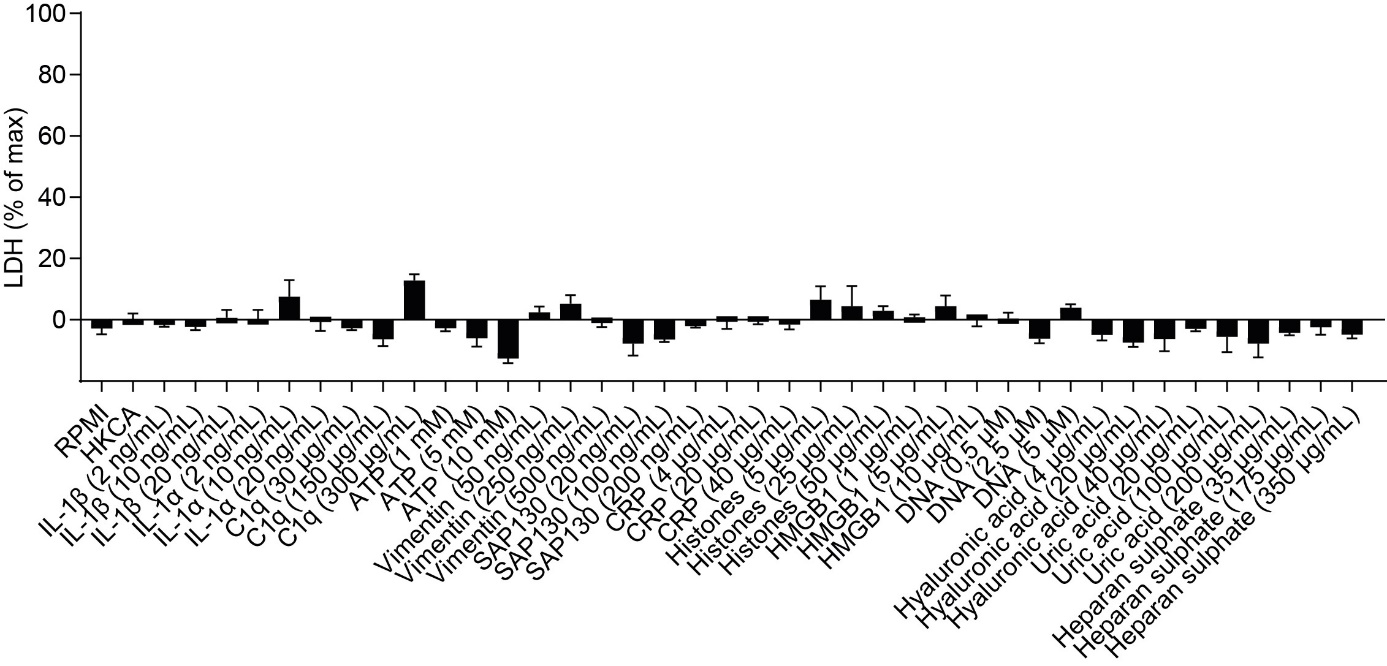 |
| --- |
| **Supplementary figure 11: DAMPs and inflammatory cytokines toxicity assay** Lactate dehydrogenase (LDH) measurement of PBMCs treated with DAMPs and inflammatory cytokines for 24 hours in three concentrations (n=3). Data are expressed as Mean ± SEM. |

| 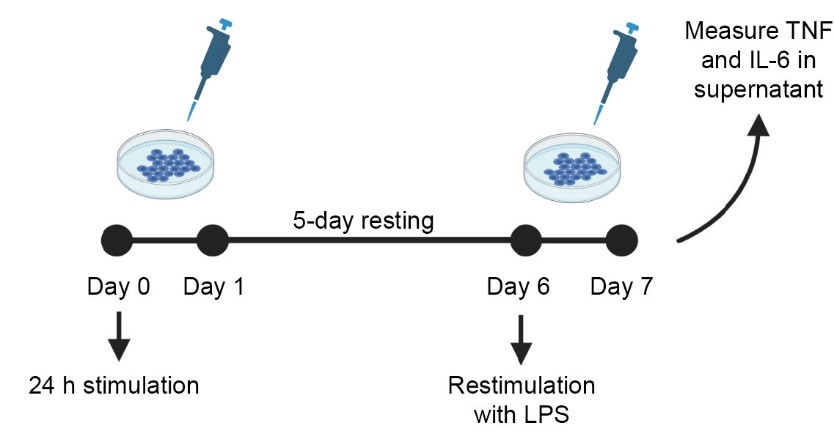 |
| --- |
| **Supplementary figure 12: Schematic representation of the trained immunity assay** Schematic representation of the trained immunity assay where PBMCs were stimulated for 24 hours with DAMPs and inflammatory cytokines in three concentrations. After a five-day resting period, cells were restimulated with LPS for 24 hours and cytokine production was measured in the supernatant by ELISA. |

| 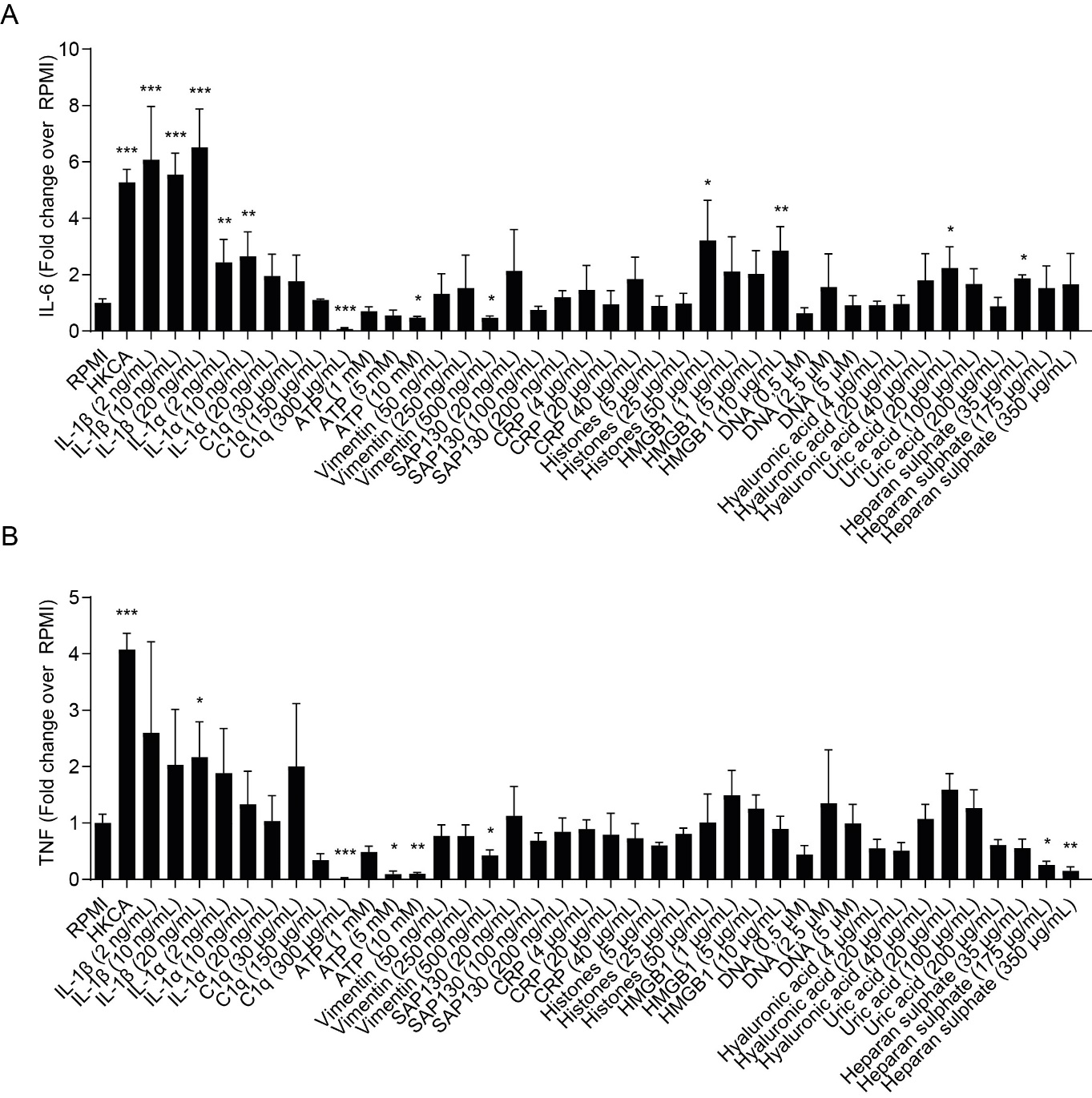 |
| --- |
| **Supplementary figure 13: DAMPs and inflammatory cytokines innate immune response A, B.** PBMCs were stimulated for 24 hours with DAMPs and inflammatory cytokines in three concentrations. After a five-day resting period, cells were restimulated with LPS for 24 hours and IL-6 and TNF cytokine production was measured in the supernatant by ELISA (n=6). Data are expressed as mean fold change over RPMI ± SEM. p-values were calculated using an unpaired t-test. * p<0.05, ** p<0.01, *** p<0.001. |

| 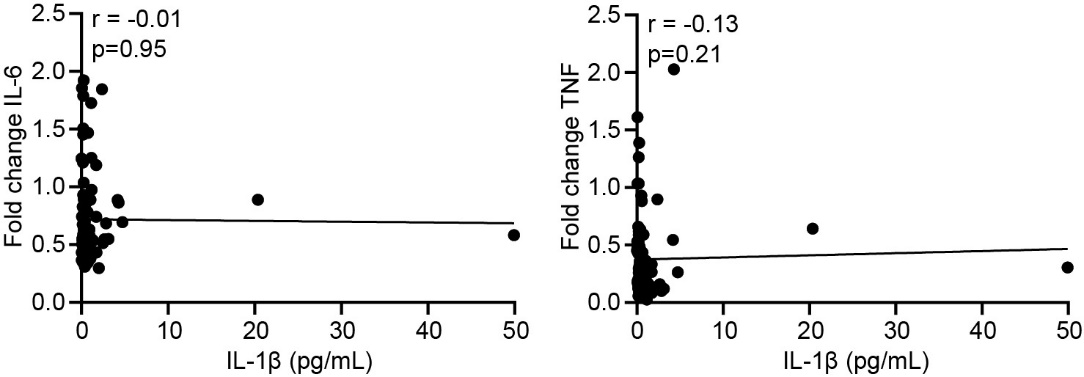 |
| --- |
| **Supplementary figure 14: No correlation between post-transplantation serum IL-1β levels and PBMC post-transplantation serum IL-6 and TNF response** Correlation between post-transplantation serum IL-1β levels and PBMC post-transplantation serum IL-6 response of 96 kidney transplant recipients |

| 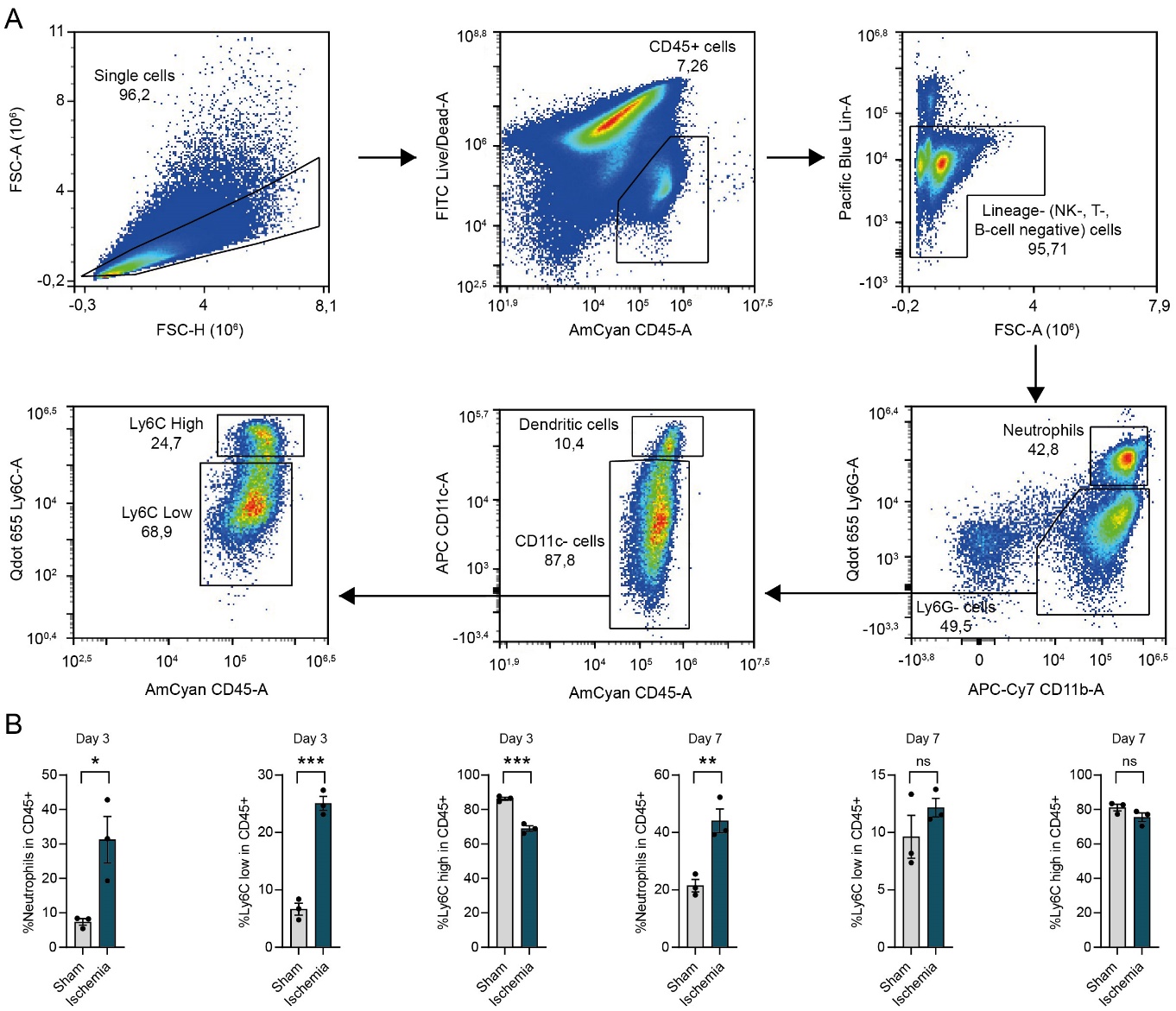 | |
| --- | --- |
| **Supplementary figure 15: The effects of ischemia reperfusion injury on the infiltration of immune cells in the kidney in mice sacrificed at day 3 or day 7  A.** Flow cytometry gating strategy for quantifications of CD45^+^, neutrophils, Ly6C low, and Ly6C high cells. Unilateral renal ischemia was induced in eight-to-twelve-week-old C57BIL6J mice for 40 minutes, sham mice underwent the same procedure without clamping of the renal artery. Mice were sacrificed on day 3 or day 7, and kidneys were harvested. Half of the kidney was cut in pieces and digested to gain a single cell suspension, and used for flow cytometric analysis.  **B.** Percentages of neutrophils, Ly6C high and Ly6C low in CD45^+^ cells. Data are expressed as Mean ± SEM. p-values were calculated using an unpaired t-test. * p<0.05, ** p<0.01, *** p<0.001. | |
| 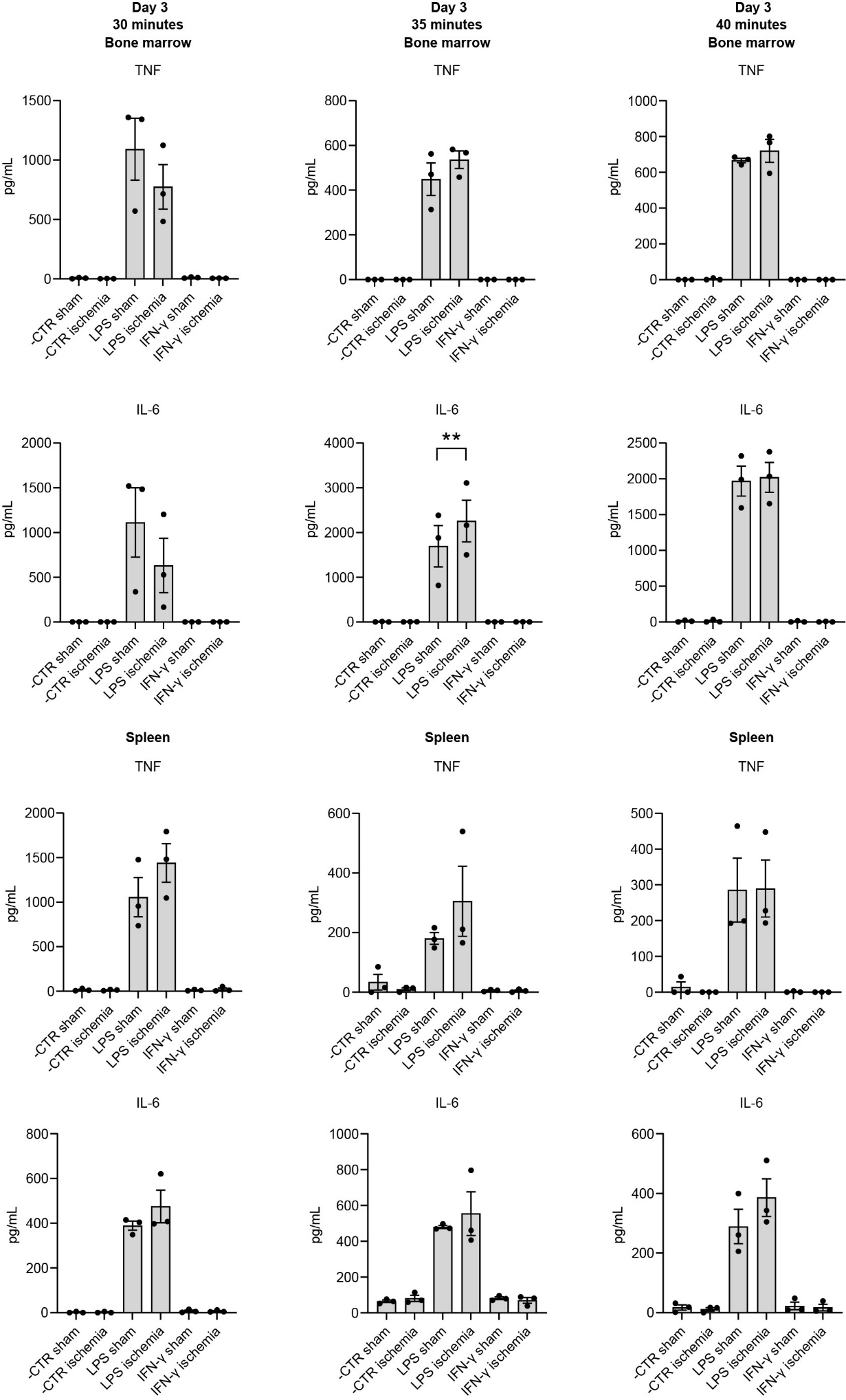 |  |
| **Supplementary figure 16: The effects of ischemia reperfusion injury on trained immunity in mice sacrificed at day 3** Unilateral renal ischemia was induced in eight-to-twelve-week-old C57BL/6J mice for 30, 35 or 40 minutes, sham mice underwent the same procedure without clamping of the renal artery. Mice were sacrificed on day 3, and bone marrow derived macrophages (BMDM) and splenocytes were collected and stimulated with LPS, IFN-γ or with culture medium (DMEM:HAMF12) as a negative control (-CTR) for 24 hours and supernatant was collected. IL-6 and TNF cytokine production was measured by ELISA (n=3). Data are expressed as Mean ± SEM. p-values were calculated using a paired t-test. |  |

| 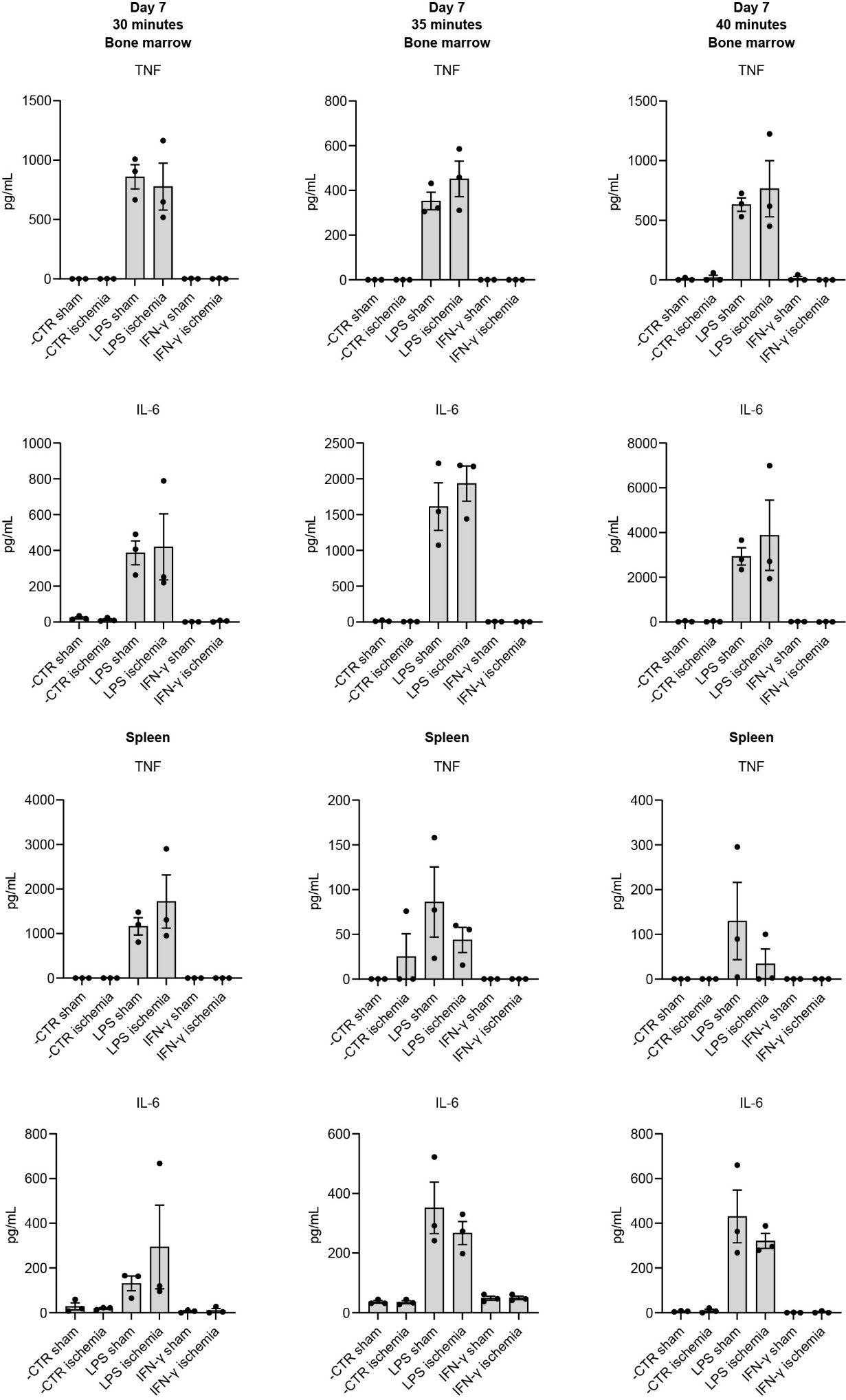 |
| --- |

| **Supplementary figure 17: The effects of ischemia reperfusion injury on trained immunity in mice sacrificed at day 7** Unilateral renal ischemia was induced in eight-to-twelve-week-old C57BL/6J mice for 30, 35 or 40 minutes, sham mice underwent the same procedure without clamping of the renal artery. Mice were sacrificed on day 7, and BMDM and splenocytes were collected and stimulated with LPS, IFN-γ or with culture medium (DMEM:HAMF12) as a negative control (-CTR) for 24 hours and supernatant was collected. IL-6 and TNF cytokine production was measured by ELISA (n=3). Data are expressed as Mean ± SEM. p-values were calculated using a paired t-test. |
| --- |

| 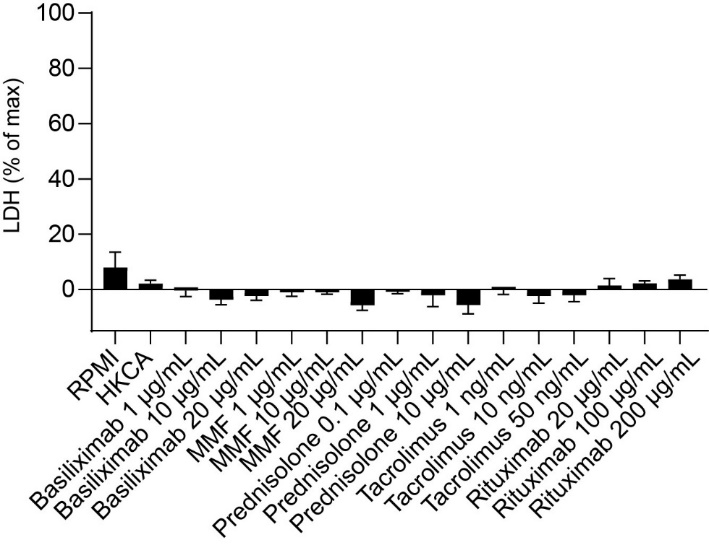 |
| --- |
| **Supplementary figure 18: Immunosuppressive drugs toxicity assay** Lactate dehydrogenase (LDH) measurement of PBMCs treated with immunosuppressive drugs for 24 hours in three concentrations (n=3). Data are expressed as Mean ± SEM. |

| 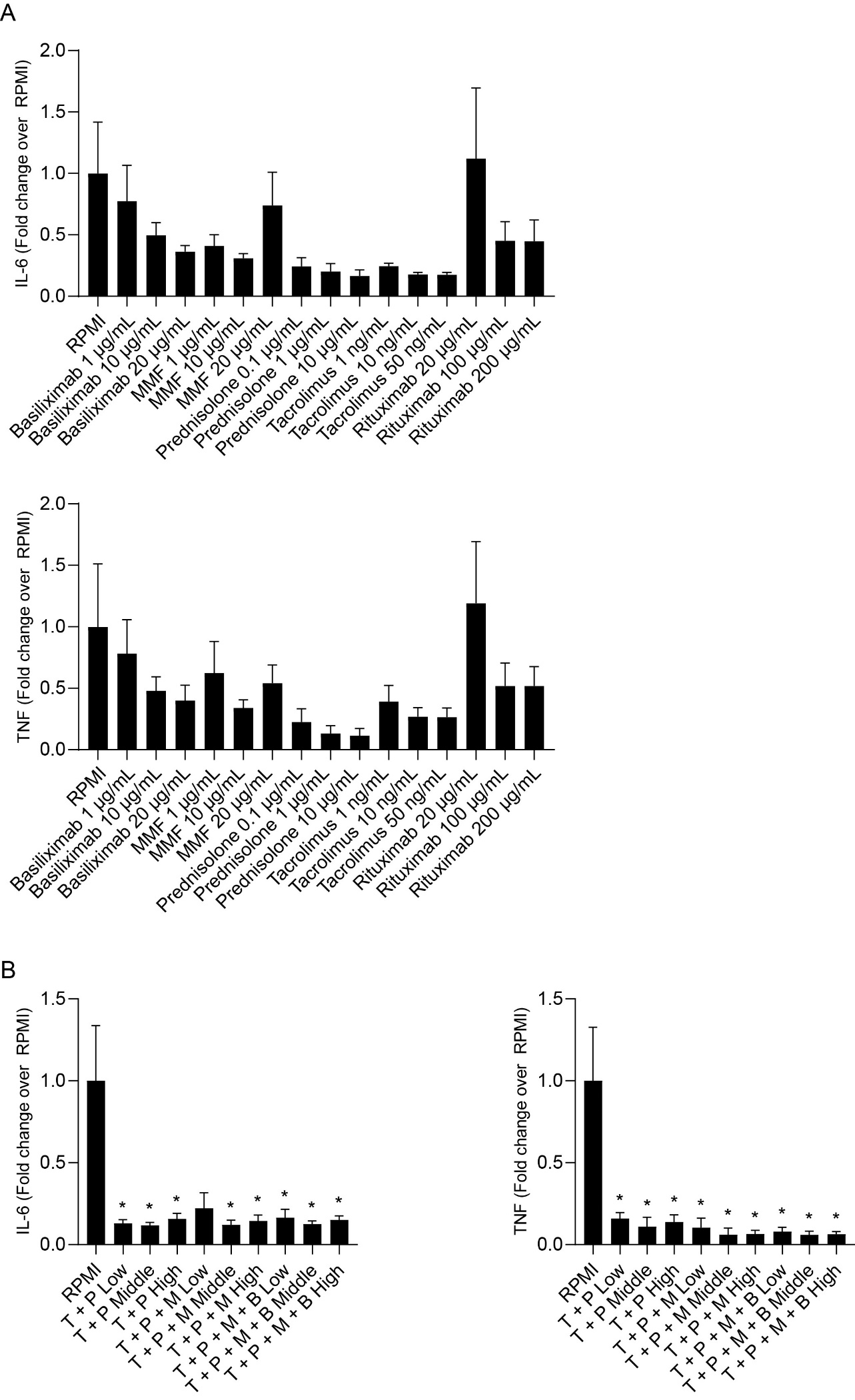 |
| --- |
| **Supplementary figure 19. Immunosuppressive drug combinations induce tolerization A.** PBMCs were incubated with immunosuppressive drugs in three dosages, for 24 hours. After 24 hours the immunosuppressive drugs were washed away. After a five-day resting period, cells were restimulated with LPS for 24 hours, and cytokine production was measured in the supernatant (n=6).  **B.** PBMCs were incubated with a combination of two, three, or four immunosuppressive drugs in three dosages (with RPMI as control) for 24 hours. After 24 hours the immunosuppressive drugs were washed away. After a five-day resting period, cells were restimulated with LPS for 24 hours, and cytokine production was measured in the supernatant (n=6). T=Tacrolimus, P = Prednisolone, M = MMF, B = Basiliximab. Low = tacrolimus 1 ng/mL, prednisolone 0.1 µg/mL, MMF 1 µg/mL, basiliximab 1 µg/mL. Middle = tacrolimus 10 ng/mL, prednisolone 1 µg/mL, MMF 10 µg/mL, basiliximab 10 µg/mL. High = tacrolimus 50 ng/mL, prednisolone 10 µg/mL, MMF 20 µg/mL, basiliximab 20 µg/mL Data are expressed as fold change compared to untrained (RPMI) PBMCs. *p < 0.05, **p < 0.01, ***p < 0.001. p-values were calculated using an unpaired t-test. |

| 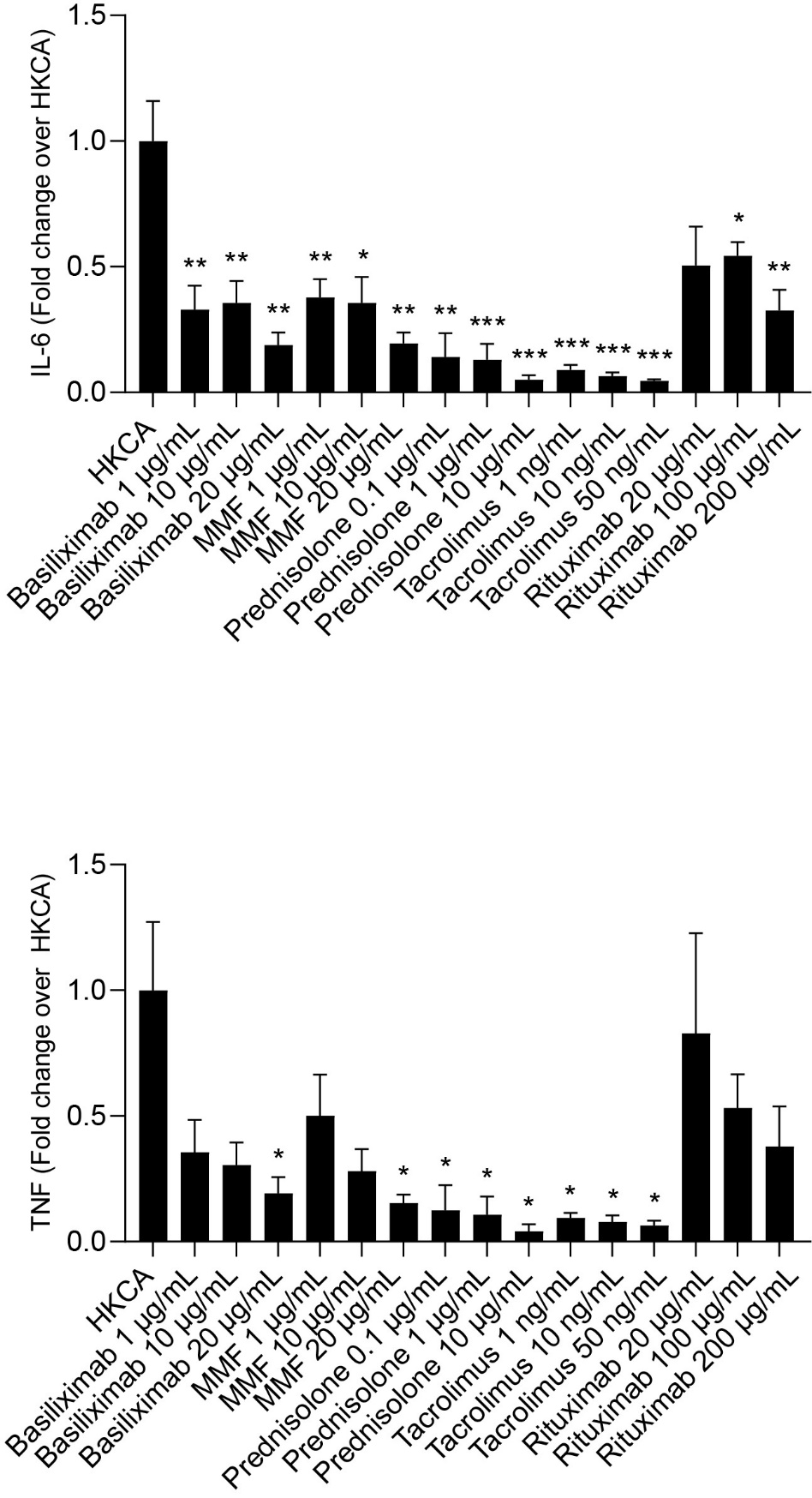 |
| --- |
| **Supplementary figure 20: Immunosuppressive drugs inhibit the trained immunity response to HKCA** PBMCs were stimulated with HKCA together with immunosuppressive drugs in different concentrations for 24 hours. After 24 hours the stimulus and immunosuppressive drugs were washed away After a five-day resting period, cells were restimulated with LPS for 24 hours and IL-6 and TNF cytokine production was measured in the supernatant by ELISA (n=6). Data are expressed as Mean fold change over HKCA ± SEM. p-values were calculated using an unpaired t-test. * p<0.05, ** p<0.01, *** p<0.001. |

| **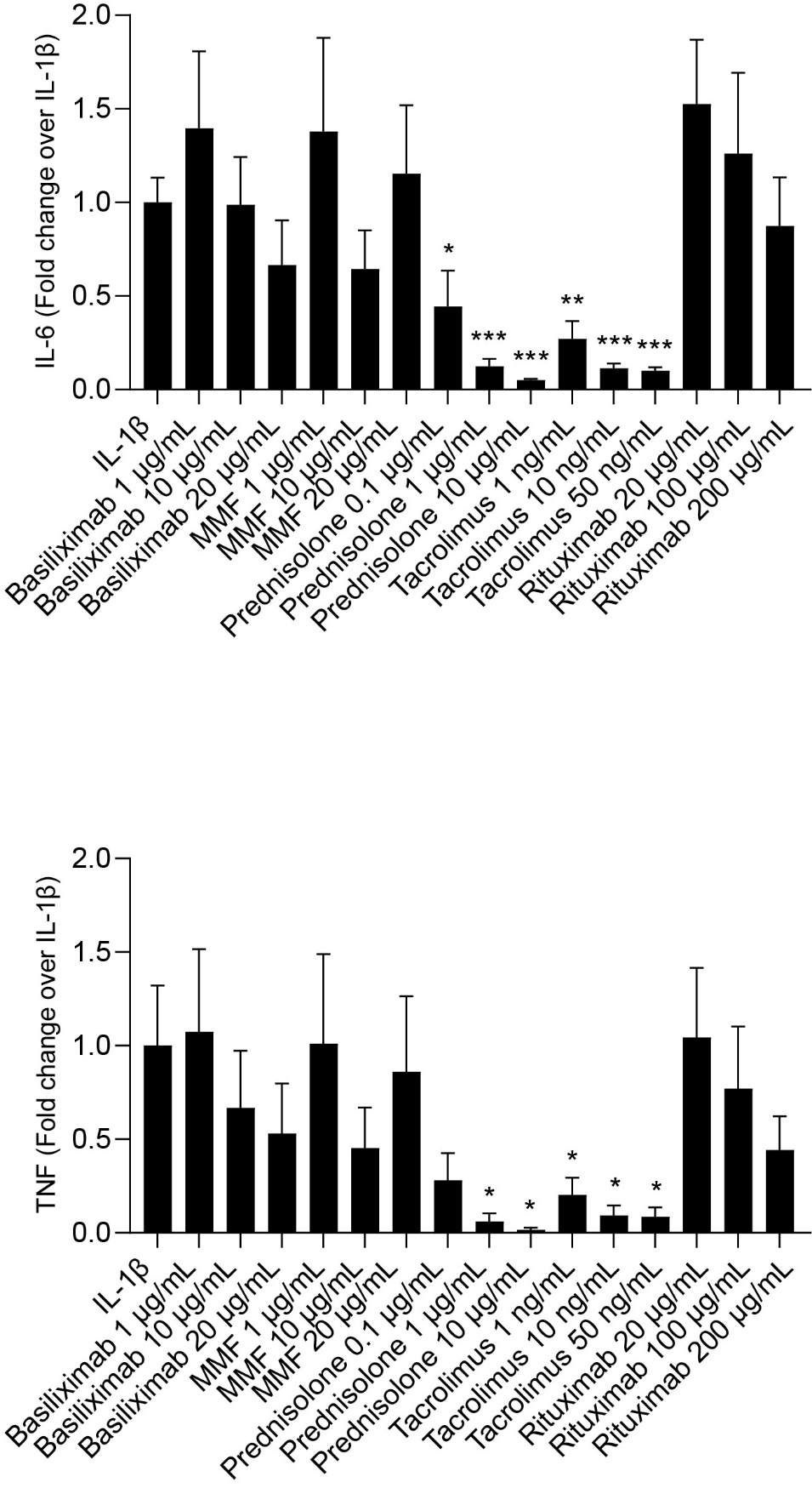** |
| --- |
| **Supplementary figure 21: Immunosuppressive drugs inhibit the trained immunity response to IL-1β** PBMCs were stimulated with HKCA together with immunosuppressive drugs in different concentrations for 24 hours. After 24 hours the stimulus and immunosuppressive drugs were washed away After a five-day resting period, cells were restimulated with LPS for 24 hours and IL-6 and TNF cytokine production was measured in the supernatant by ELISA (n=6). Data are expressed as Mean fold change over IL-1β ± SEM. p-values were calculated using an unpaired t-test. * p<0.05, ** p<0.01, *** p<0.001. |

| 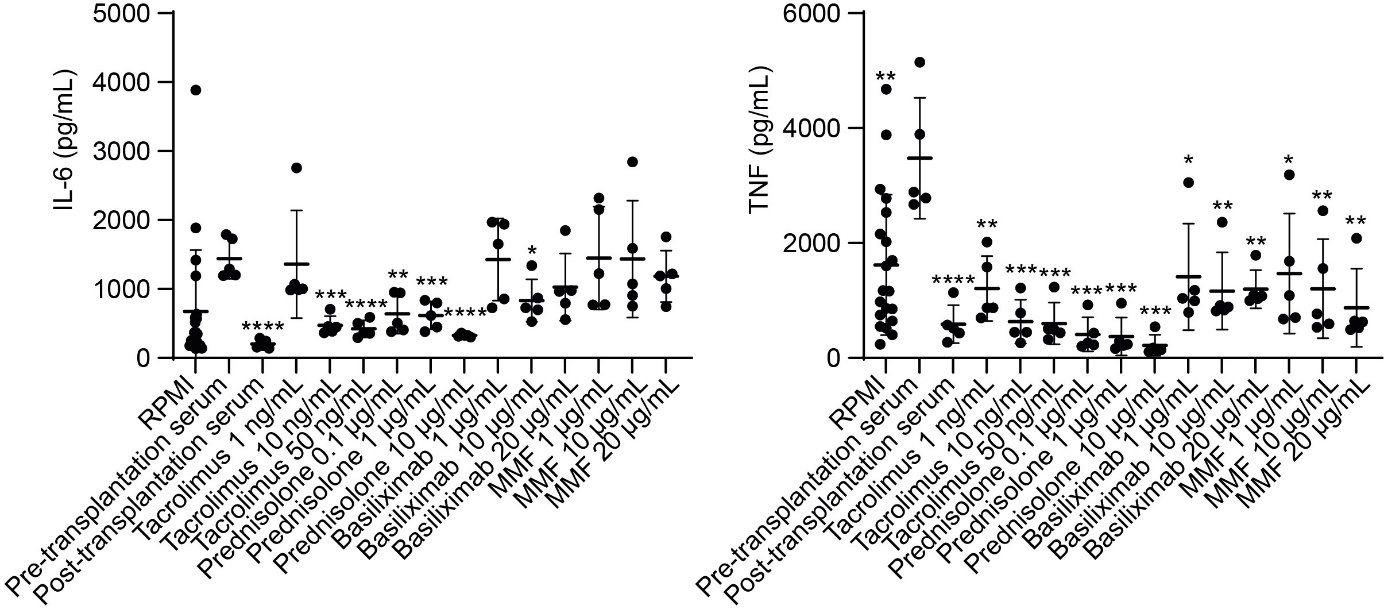 |
| --- |
| **Supplementary figure 22: Immunosuppressive drugs inhibit the trained immunity response to pre-transplant serum** PBMCs were stimulated with post-transplant serum, pre-transplant serum or pre-transplant serum together with immunosuppressive drugs in different concentrations for 24 hours, RPMI acted as control. After 24 hours the stimulus and immunosuppressive drugs were washed away After a five-day resting period, cells were restimulated with LPS for 24 hours and IL-6 and TNF cytokine production was measured in the supernatant by ELISA. Data are expressed as Mean ± SD. p-values were calculated using an unpaired t-test. p-values demonstrated were calculated by comparing conditions to pre-transplant serum. * p<0.05, ** p<0.01, *** p<0.001, **** p<0.0001. |

| 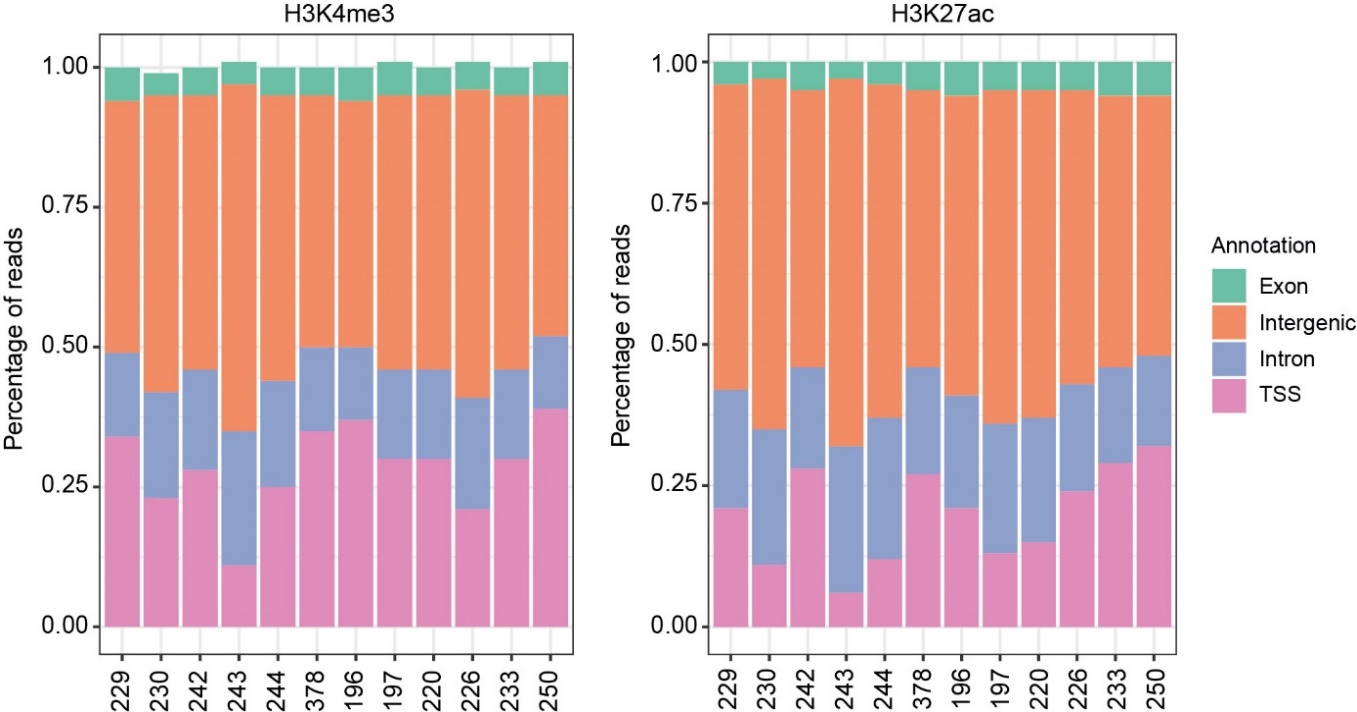 |
| --- |
| **Supplementary figure 23: Genomic annotations in patients from lowest and highest tertile of post-transplant serum induced trained immunity** Genomic annotations of H3K4me3 and H3K27ac peaks in individual samples of 6 patients from the lowest tertile (229, 230, 242, 243, 244, 378), and 6 patients from the highest tertile (196, 197, 220, 226, 233, 250). |

| 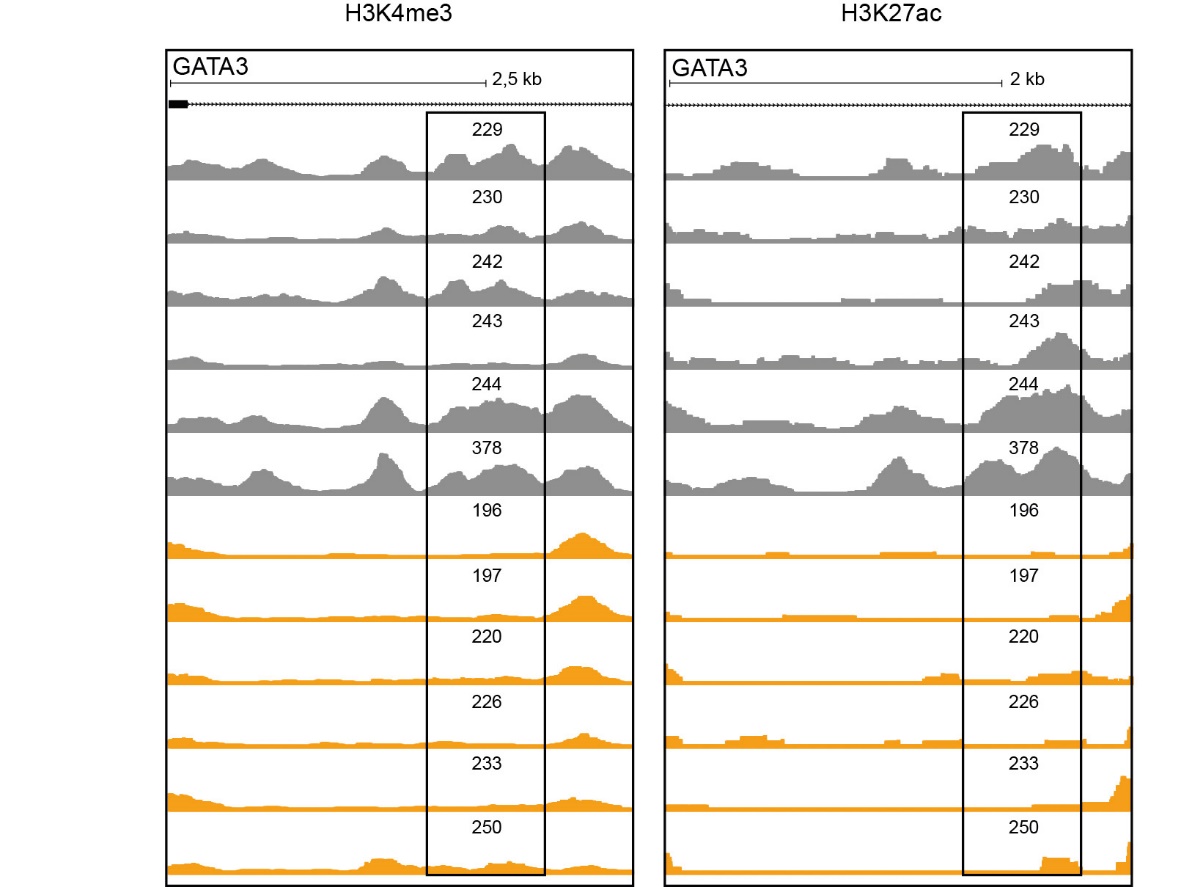 |
| --- |
| **Supplementary figure 24:** **Epigenetic profiles of kidney transplant recipient’s circulating leukocytes** H3K4me3 and H3K27ac signal at GATA3 gene as visualized in the UCSC genome browser. 6 patients from the lowest tertile (229, 230, 242, 243, 244, 378, grey), and 6 patients from the highest tertile (196, 197, 220, 226, 233, 250, orange) |

**DATA AND MATERIAL AVAILABILITY**

| **Reagent or Resource** | **Source** | **Identifier** |
| --- | --- | --- |
| **Antibodies** | | |
| Anti-Mouse CD90.2 (Thy-1.2) eFluor450 | Thermo Fisher Scientific | Cat#48-0902-82 (RRID: AB_1272200) |
| Anti-Mouse Ter119 eFluor450 | Thermo Fisher Scientific | Cat#48-5921-82 (RRID: AB_1518808) |
| Anti-Mouse NK1.1 eFluor450 | Thermo Fisher Scientific | Cat#48-5941-82 (RRID: AB_2043877) |
| Anti-Mouse CD49b (Integrin alpha 2) eFluor450 | Thermo Fisher Scientific | Cat#48-5971-82 (RRID: AB_10671541) |
| Anti-Human/Mouse CD45R(B220) eFluor450 | Thermo Fisher Scientific | Cat#48-0452 (RRID: AB_10422068) |
| Brilliant Violet 510 Anti-Mouse CD45 | Biolegend | Cat#103137 (RRID: AB_2561392) |
| Brilliant Violet 785 Anti-Mouse Ly6C | Biolegend | Cat#128041 (RRID: AB_2565852) |
| APC/Cyanine7 Anti-Mouse/Human CD11b | Biolegend | Cat#101225 (RRID: AB_830641) |
| APC Anti-Mouse CD11c | Biolegend | Cat#117309 (RRID: AB_313778) |
| PE/Cyanine7 Anti-Mouse F4/80 | Biolegend | Cat#123113 (RRID: AB_893490) |
| Brilliant Violet 650 Anti-Mouse Ly-6G | Biolegend | Cat#127641 (RRID: AB_2565881) |
| **Biological Samples** | | |
| Human PBMCs from buffy coats | Sanquin blood bank | Cat#B2825R00 |
| **Chemicals, Peptide and Recombinant Proteins** | | |
| Ficoll-Pague (Lymphoprep) | StemCell Technologies, Inc. | Cat#07861 |
| Glutamax | Thermo Fisher Scientific | Cat#35050 |
| Pyruvate | Thermo Fisher Scientific | Cat#11360 |
| Penicilin/Streptomycin | Thermo Fisher Scientific | Cat#15140 |
| RPMI 1640 | Thermo Fisher Scientific | Cat#22409 |
| Fetal Bovine serum | Serana Europe GmbH | Cat#S-FBS-EU-025 |
| Heat-Killed Candida albicans | Invivogen | Cat#tlrl-hkca |
| Lipopolysaccharide-B5 Ultrapure | Invivogen | Cat#tlrl-pb5lps |
| Lipopolysaccharide from Pseudomonas aeruginosa | Sigma-Aldrich | Cat#L9143 |
| Recombinant Human Vimentin Protein | Novusbio | Cat#NBP2-35139 |
| IL-1 alpha/IL-1F1 Protein | Novusbio | Cat#NBP2-35051 |
| Recombinant Human SAP130 Protein | Novusbio | Cat#H00023450-Q01 |
| ATP | Invivogen | Cat#tlrl-atpl |
| ODN 2009 (ODN 7909) | Invivogen | Cat#tlrl-2006 |
| Uric acid | Sigma-Aldrich | Cat#U2625 |
| Complement C1q, human | Calbiochem | Cat#204876 |
| Recombinant Human C-Reactive Protein/CRP | R&D systems | Cat#1707-CR |
| Recombinant human IL-1β | Invivogen | Cat#rcyec-hillb |
| Hyaluronic Acid Oligosaccharide | Iduron | Cat#HA06 |
| Heparan sulfate sodium salt from bovine kidney | Sigma-Aldrich | Cat#H7640 |
| HMGB1 | R&D systems | Cat#1690-HMB |
| Simulect 10 mg (Basiliximab) | Novartis | - |
| Tacrolimus | Sigma-Aldrich | Cat#F4679 |
| Prednisolone | Sigma-Aldrich | Cat#P6004 |
| CellCept 500 mg (mycofenolaatmofetil) | Roche | - |
| Truxima 10 mg/mL (Rituximab) | Celltrion Healthcare | - |
| 16% Formaldehyde | Sigma-Aldrich | Cat#28908 |
| Protease Inhibitor Cocktail (tablets) | Roche | Cat#04693132001 |
| DNase I | Qiagen | Cat#79254 |
| Lysis buffer: BD Pharm Lyse™ | BD Biosciences | Cat# 555899 |
| DNase I, grade II | Roche | Cat#10104159001 |
| Collagenase IV | Sigma-Aldrich | Cat#C5138 |
| Mouse IFN-gamma Recombinant Protein | R&D systems | Cat#485MI |
| DMEM:HAMF12 | Thermo Fisher Scientific | Cat#21041-025 |
| BD FC block | BD Bioscience | Cat#553142 |
| Cell Staining Buffer | Biolegend | Cat#420201 |
| **Critical Commercial Assays** | | |
| Human IL-6 Duoset ELISA | R&D systems | Cat#DY206 |
| Human TNF-α Duoset ELISA | R&D systems | Cat#DY210 |
| Mouse IL-6 Duoset ELISA | R&D systems | Cat#DY406 |
| Mouse TNF- α Duoset ELISA | R&D systems | Cat#DY410 |
| Simple Plex Cartridge Kit for 32 samples, containing IL-1α, IL-1β, IL-6, TNF-α for use with Human Plasma/ Serum | Bio-techne | Cat#SPCK-PS-000446 |
| Human IL-1β control | Bio-techne | Cat#894962 |
| Human IL-6 control | Bio-techne | Cat#894968 |
| Human IL-1α control | Bio-techne | Cat#898055 |
| Human TNF-α control | Bio-techne | Cat#894977 |
| Sample Diluent SD13 | Bio-techne | Cat#992517 |
| CyQuant LDH Cytotoxicity Assay | Thermo Fisher Scientific | Cat#C20301 |
| RNeasy Mini kit | Qiagen | Cat#74106 |
| MagnaChIP kit | Merck-Millipore | Cat#17-408 |
| MinElute Reaction Cleanup Kit | Qiagen | Cat#28204 |
| LIVE/DEAD Fixable Green Dead Cell Stain Kit, for 488 nm excitation | Thermo Fisher Scientific | Cat#L34970 |
| FITC Annexin V Apoptosis Detection Kit with PI | Biolegend | Cat#640914 |
| **Software** | | |
| IBM SPSS Statistics 25 | IBM® SPSS® Statistics Software | N/A |
| GraphPad Prism | Graphpad software | N/A |
| R | R core Team | <https://www.r-project.org/> |
| Hisat | Kim et al.^1^ | http://www.ccb.jhu.edu /software/hisat/index.shtml |
| Samtools | Li et al.^2^ | <http://samtools.source>forge.net |
| DESeq2 | Love et al.^3^ | http://www.bioconductor.org/ packages/release/bioc/ html/DESeq2.html |
| ggplot2 | Wickham^4^ | https://ggplot2.tidyverse.org/ |
| Complex Heatmaps | Gu et al.^5^ | <http://www.bioconductor.org/> packages/devel/bioc/html/ ComplexHeatmap.html |
| fgsea R package | Subramanian et al.^6^ | <https://bioconductor.org/> packages/release/bioc/html/fgsea.html |
| Burros Wheeler Aligner | Li et al.^7^ | http://maq.sourceforge.net/ |
| Model-based analysis of ChIP-Seq | Zhang et al.^8^ | <http://liulab.dfci.harvard>.edu/MACS/ |
| BEDtools | Quinlan et al.^9^ | <http://code.google.com/p/>bedtools |
| Great | McLean^10^ | <http://great.stanford.edu/%20public> |
| NovoExpress software 1.5.6 | Agilent | N/A |
| 1. Kim D, Langmead B, Salzberg SL. HISAT: a fast spliced aligner with low memory requirements. Nature methods. 2015;12(4):357-360.  2. Li H, Handsaker B, Wysoker A, et al. The sequence alignment/map format and SAMtools. Bioinformatics. 2009;25(16):2078-2079.  3. Love MI, Huber W, Anders S. Moderated estimation of fold change and dispersion for RNA-seq data with DESeq2. Genome biology. 2014;15(12):1-21.  4. Wickham H. Elegant graphics for data analysis. Media. 2009;35(211):10.1007.  5. Gu Z, Eils R, Schlesner M. Complex heatmaps reveal patterns and correlations in multidimensional genomic data. Bioinformatics. 2016;32(18):2847-2849.  6. Subramanian A, Tamayo P, Mootha VK, et al. Gene set enrichment analysis: a knowledge-based approach for interpreting genome-wide expression profiles. Proceedings of the National Academy of Sciences. 2005;102(43):15545-15550.  7. Li H, Durbin R. Fast and accurate short read alignment with Burrows–Wheeler transform. bioinformatics. 2009;25(14):1754-1760.  8. Zhang Y, Liu T, Meyer CA, et al. Model-based analysis of ChIP-Seq (MACS). Genome biology. 2008;9(9):1-9.  9. Quinlan AR, Hall IM. BEDTools: a flexible suite of utilities for comparing genomic features. Bioinformatics. 2010;26(6):841-842.  10. McLean CY, Bristor D, Hiller M, et al. GREAT improves functional interpretation of cis-regulatory regions. Nature biotechnology. 2010;28(5):495-501. | | |
