# Supplementary material for: Trained immunity suppression determines kidney allograft survival
Source: Am J Transplant. Author manuscript; Available in PMC 2025 Nov 1. (PMC11789421; doi:10.1016/j.ajt.2024.08.006)
Supplement: Multimedia component 2 [file NIHMS2046486-supplement-Multimedia_component_2.docx]

**SUPPLEMENTARY MATERIALS AND METHODS**

**Human subjects**

For in vitro studies on human PBMCs, buffy coats from healthy donors were obtained from Sanquin blood bank, Nijmegen after written consent, from which no additional details are available. Serum and PBMCs of transplant patients collected before and one week after kidney transplantation were obtained from patients included in a randomized controlled trial conducted at Radboud University Medical Center (1, 2). Patients were included when there was serum available pre-transplantation and one week post-transplantation, and PBMCs one week post-transplantation, which resulted in a cohort of 96 patients.

**PBMC isolation**

PBMCs were isolated by differential centrifugation over Ficoll-Pague (Lymphoprep, Stemcell Technologies). Cells were washed three times in PBS, and PBMCs were resuspended in RPMI 1640 culture medium supplemented with 2mM glutamax, 1mM pyruvate, and penicillin/streptomycin (all from Thermo Fisher Scientific) and counted on a Casy cell counter.

**Trained immunity assays**

Human PBMCs were plated in 96-well flat bottom plates, with 500.000 cells per well. Cells were left to adhere for 1 hour at 37°C. After one hour cells were washed 3 times with PBS. After washing PBMCs were incubated with culture medium supplemented with 10% FBS, only as a negative control, HKCA, 10^5^ cells/mL (Invivogen), that acted as a positive control, a first stimulus (DAMP, immunosuppressive drug, 10% patient serum) alone, or a first stimulus in combination with HKCA or IL-1β (Invivogen) for 24 hours at 37°C. After 24 hours cells were washed to remove soluble stimuli. It cannot be excluded that compounds bound to the cells remain present in the culture after washing. Cells were rested for five days in culture medium containing 10% FBS. After the resting period cells were stimulated with either RPMI culture medium as a negative control, or 10 ng/mL LPS, (Invivogen) for 24 hours. After 24 hours supernatant was collected.

**Lactate dehydrogenase measurements to assess cellular toxicity**

Lactate dehydrogenase (LDH) concentration was measured in supernatants of PBMCs after 24-hour incubation with DAMPs using the CyQuant LDH Cytotoxicity Assay (Thermo Fisher Scientific). LDH concentration was calculated as percentage of the maximal possible LDH concentration in completely lysed cells according to formula:

$LDH \left( \% of max \right)= \left( \frac{Compound induced LDH-Spontaneous LDH}{Maximum LDH-Spontaneous LDH} \right) x 100$.

**PI and Annexin V staining for determination of cell death**

PBMCs were seeded in 12 wells plates in a density of 5*10^6^ cells/well, washed twice with PBS and incubated with 10% pre-transplant serum or RPMI as control in culture medium supplemented with 10% FBS for 24 hours. Cells were washed with Cell Staining buffer (Biolegend) and incubated in Versene solution (0.48mM EDTA in PBS) for 30 min. Cells were scraped in Versene and counted using a Casy counter. For each sample, 1*10^5^ cells were stained using the FITC Annexin V Apoptosis Detection Kit with PI (Biolegend), according to manufacturer’s instructions. PBMCs that were incubated for 2 h in 1 µg/mL staurosporine solution from *Streptomyces* sp. (Sigma Aldrich) were included as positive control. FITC Annexin V and PI staining was detected with an ACEA Novocyte 3000 (Agilent).

**Cytokine/protein measurements**

Cytokine production was measured at day 7 in supernatants using commercial ELISA kits for human TNF and IL-6 (R&D systems) according to manufacturer’s instructions.

Inflammation-related protein biomarkers were measured by Olink Proteomics, with the Inflammation panel (Olink Proteomics, Uppsala Sweden). This panel can measure relative levels of 92 inflammation-related protein biomarkers. Protein levels are shown in Normalized Protein eXpression (NPX) values on a log2 scale. Proteins were excluded when over 50% of the samples were under the limit of detection (LOD).

**RNA isolation, library preparation and sequencing for transcriptomic analysis**

For RNA isolation 1*10^6^ PBMCs were resuspended in 350 uL of RNA later Buffer (QIAGEN). RNA was isolated using RNeasy kit (QIAGEN) including deoxyribonuclease (DNase)I (QIAGEN) digestions. RNA bulk sequencing was performed by Single Cell Discoveries (Utrecht, The Netherlands) with a sequencing depth of 20 million reads/sample. Library preparation was performed according to the CEL-seq2 protocol (3). Sequencing was performed on a Nextseq500 (Illumina).

**Chromatin Immunoprecipitation**

PBMCs were resuspended in RPMI culture medium and fixed using formaldehyde (1% final concentration, Sigma Aldrich) for 10 minutes at room temperature. Unreacted formaldehyde was quenched with 125 mM glycine and incubated for 5 minutes at room temperature. Cells were washed twice in PBS containing protease inhibitor cocktail (Roche) and 1 mM PMSF (Roche), and subsequently snap frozen in liquid nitrogen. Cell pellets were stored at -80°C for further use. Cells were sonicated at a concentration of 15 million cells/mL using a Bioruptor pico sonicator (Diagenode; 10-20 cycles, 30s on, 30s off, at 4°C. Immunoprecipitation was performed using the MagnaChIP kit (Merck Millipore) according to manufacturer’s instruction. In short, 500.000 cells were incubated overnight with 1 mg H3K4me3 or H3K27ac (Diagenode) and protein A magnetic beads at 4°C. Beads and chromatin/antibody mixture were washed four times for 5 minutes at 4°C. After washing chromatin was eluted and proteins were degraded using proteinase K. DNA was purified using spin columns and eluted in millliQ.

**Library preparation and sequencing of ChIP samples**

ChIP-seq libraries were prepared using the Kapa Hyper Prep Kit according to manufacturer’s protocol, with the following modifications. 2.5 mL of the NEXTflex adaptor stock (600 nM, Bio Scientific) was used for adaptor ligation of each sample. Libraries were amplified with 12-15 PCR cycles followed by a double post-amplification clean-up was used to ensure proper removal of adapters. Samples were analyzed for purity using a High Sensitivity DNA Chip on a Bioanalyzer 2100 system (Agilent). Libraries were paired-end sequenced to a read length of 50 bp on an Illumina NextSeq500.

**Mouse renal ischemia-reperfusion injury model**

**Animals**

Eight-to-twelve-week-old C57BL/6J female mice (Charles River, Germany) were kept under pathogen-free conditions and housed in a temperature-controlled room with a 12-hour light/dark cycle with ad libitum access to food and water. All experiments were approved by the Animal Ethics Committee of the Radboud University Nijmegen.

**Ischemia-reperfusion injury model**

Eight-to-twelve-week-old C57BL/6J mice were anesthetized with a ketamine (75 mg/kg) -dexmedetomidine (0.5 mg/kg) intraperitoneal injection. Renal ischemia in mice was established by unilaterally clamping the renal artery for 40 minutes, sham mice underwent the same procedure, without clamping the renal artery. Mice were placed on a heating pad to regulate body temperature and body temperature was monitored trough a rectal probe. Pain relief was provided with buprenorphine for 48 hours after surgery or until pain symptoms disappeared. After either three or seven days mice were euthanized with CO_2_, and blood, ischemic/sham kidney, spleen and femurs were collected.

**Flow cytometry**

Half of the kidney, spleen, blood, and femurs were collected after euthanizing the mice, and used for flow cytometry analysis. Kidneys were cut in pieces and put in an enzymatic digestion solution containing 0.2 mg/mL collagenase IV (Sigma Aldrich) and 0.05 mg/mL DNaseI (Sigma Aldrich) and placed in a 37 °C incubator for 1 hour. Cells were forced through a 70 μm strainer and resuspended in Ammonium-Chloride-Potassium (ACK) lysis buffer (155 mM NH_4_Cl, 10 mM KHCO_3_, 0.1 mM EDTA) for 5 minutes at room temperature. The spleen was mashed and forced through a 70 μm strainer. Subsequently blood, spleen, and kidney cells were lysed with BD Pharm Lyse (BD Bioscience) for 4 minutes at room temperature. For the collection of bone marrow cells, both ends of the femur were cut with sterile scissors and a 23-gauge needle was used to flush the bone marrow out of the femur. Isolated bone marrow cells were run through a 70 μm strainer and counted on a Casy cell counter.

For flow cytometric staining 4x10^6^ kidney cells, 2x10^6^ spleen cells, 1x10^6^ bone marrow cells, and 1x10^6^ blood cells were used per sample. Fc receptors on cells were blocked with mouse BD FC block (BD Bioscience). The following antibodies were used: Brilliant Violet 510 anti-mouse CD45 antibody (Biolegend), Brilliant Violet 785 anti-mouse Ly-6C antibody (Biolegend), APC/Cyanine7 anti-mouse/human CD11b antibody (Biolegend), APC anti-mouse CD11c antibody (Biolegend), PE/Cyanine7 anti-mouse F4/80 antibody (Biolegend) Brilliant Violet 650 anti-mouse Ly-6G antibody (Biolegend), eFluor450 anti-mouse CD49b antibody (Thermo Fisher Scientific), eFluor450 anti-mouse CD90.2 antibody (Thermo Fisher Scientific), eFluor450 anti-mouse Nk1.1 antibody (Thermo Fisher Scientific), eFluor450 anti-mouse Ter119 antibody (Thermo Fisher Scientific). The antibody dilutions ranged from 1:200 to 1:100 unless stated otherwise. Flow cytometry was performed with the NovoSampler Pro (Acea Biosciences, San Diego, USA), and data were analyzed in the NovoExpress software version 1.5.6 (Acea Biosciences).

**Cell culture**

Bone marrow derived macrophages (BMDM) and splenocytes were plated in 96-well flat bottom plates, with 500.000 cells per well. Cells were left to adhere for 1h at 37°C. After 1 hour cells were washed 3 times with PBS. After washing BMDM and splenocytes were incubated with DMEM:HAMF12 culture medium that acted as a negative control, 100 ng/mL LPS (Sigma-Aldrich), or 100 ng/mL IFN-γ (R&D systems) for 24 hours. After 24 hours supernatant was collected.

**Cytokine production**

Cytokine production of BMDMs and splenocytes was measured in culture supernatants using the commercial ELISA kits mouse Duoset IL-6 (R&D systems), and mouse Duoset TNF-α (R&D systems). The entire procedure was performed according to manufacturer’s instructions. Samples were diluted 10 times in PBA (1% BSA in PBS).

**Statistical analysis**

***In vitro* experiments data analysis**

For *in vitro* training experiments, data is shown as mean ± SEM and significance was tested with an unpaired t-test. Data was analyzed using Graphpad Prism 9.0. A p-value of less than 0.05 was considered to be statistically significant.

**Patient data analysis**

Patient data were analyzed with IBM SPSS Statistics for Windows, Version 27.0. IBM Corp. Multiple linear regression analysis with backward elimination was used to assess the association between trained immunity induced with serum obtained one week post-transplantation and the following characteristics of the recipients, donors, and patients’ age, gender, presence of hypertension, presence of diabetes, body mass index (kg/m^2^), whether the transplantation was pre-emptive, presence of donor-specific antibodies pre-transplantation, type of induction therapy, the number of HLA mismatches, type of donor (living or deceased), cold ischemia time, and the trained immunity response (IL-6 or TNF) to serum obtained pre-transplantation. Variables were retained if p < 0.05.

**RNA-seq data analysis**

Differential gene expression analysis was performed on counts of 3 samples per treatment group using the DESeq2 package in Rstudio (4). Samples were paired for donor. Differentially Expressed Genes (DEGs) were defined as fold change (FC) < 0.5 or FC > 2 and FDR < 0.1 (5).

**Gene set enrichment analysis**

Gene set enrichment analyses (GSEA) was performed on normalized counts of samples using GSEA software v4.1.0 provided by Broad Institute (6). GSEA was performed using the gene sets from HALLMARK database of the Molecular Signature database (MSigDB) (7, 8). Analyses were conducted with 1000 gene set permutations and with the following settings: Metric for ranking genes: Signal2Noise; Remap/Collapse to gene symbols: Collapse; Enrichment statistic: weighted; Normalization mode: meandiv. For each gene set, a Normalized Enrichment Score (NES) was calculated. Gene sets for which FDR < 0.1 were considered to be enriched.

**ChIP-seq data analysis**

ChIP sequencing data was aligned to human genome hg19 with BWA.(9) Samtools was used to filter reads with a quality score lower than 20, and PCR duplicates were removed with Picard (10). Peaks were identified with MACS 2.2.6 in paired-end mode and ‘call-summits’ enabled at a false discovery rate of 0.01 (11). A union of all identified peaks was generated with BEDTools, which was used to count reads per peak in each sample (12). Read counts were analyzed with DESeq2 to identify significant dynamics, as described for the RNA-seq analysis. We used GREAT to identify significantly associated gene ontologies, and to assign each ChIP peak to its closest gene for integration between ChIP- and RNA-seq data (13).

1. Joosten I, Baas M, Kamburova E, van den Hoogen M, Koenen H, Hilbrands L. Anti-B cell therapy with rituximab as induction therapy in renal transplantation. Transplant immunology. 2014;31(4):207-9.

2. van den Hoogen MW, Kamburova EG, Baas MC, Steenbergen E, Florquin S, Koenen H, et al. Rituximab as induction therapy after renal transplantation: a randomized, double-blind, placebo-controlled study of efficacy and safety. American Journal of Transplantation. 2015;15(2):407-16.

3. Hashimshony T, Senderovich N, Avital G, Klochendler A, de Leeuw Y, Anavy L, et al. CEL-Seq2: sensitive highly-multiplexed single-cell RNA-Seq. Genome Biology. 2016;17(1):77.

4. Love MI, Huber W, Anders S. Moderated estimation of fold change and dispersion for RNA-seq data with DESeq2. Genome Biology. 2014;15(12):550.

5. Szklarczyk D, Franceschini A, Wyder S, Forslund K, Heller D, Huerta-Cepas J, et al. STRING v10: protein–protein interaction networks, integrated over the tree of life. Nucleic acids research. 2015;43(D1):D447-D52.

6. Subramanian A, Tamayo P, Mootha VK, Mukherjee S, Ebert BL, Gillette MA, et al. Gene set enrichment analysis: A knowledge-based approach for interpreting genome-wide expression profiles. Proceedings of the National Academy of Sciences. 2005;102(43):15545-50.

7. Liberzon A, Birger C, Thorvaldsdóttir H, Ghandi M, Mesirov JP, Tamayo P. The Molecular Signatures Database (MSigDB) hallmark gene set collection. Cell Syst. 2015;1(6):417-25.

8. Jassal B, Matthews L, Viteri G, Gong C, Lorente P, Fabregat A, et al. The reactome pathway knowledgebase. Nucleic Acids Res. 2020;48(D1):D498-d503.

9. Li H, Durbin R. Fast and accurate short read alignment with Burrows–Wheeler transform. bioinformatics. 2009;25(14):1754-60.

10. Li H, Handsaker B, Wysoker A, Fennell T, Ruan J, Homer N, et al. The sequence alignment/map format and SAMtools. Bioinformatics. 2009;25(16):2078-9.

11. Zhang Y, Liu T, Meyer CA, Eeckhoute J, Johnson DS, Bernstein BE, et al. Model-based analysis of ChIP-Seq (MACS). Genome biology. 2008;9(9):1-9.

12. Quinlan AR, Hall IM. BEDTools: a flexible suite of utilities for comparing genomic features. Bioinformatics. 2010;26(6):841-2.

13. McLean CY, Bristor D, Hiller M, Clarke SL, Schaar BT, Lowe CB, et al. GREAT improves functional interpretation of cis-regulatory regions. Nature biotechnology. 2010;28(5):495-501.
